# Effect of Different Parameters of In Vitro Static Tensile Strain on Human Periodontal Ligament Cells Simulating the Tension Side of Orthodontic Tooth Movement

**DOI:** 10.3390/ijms23031525

**Published:** 2022-01-28

**Authors:** Changyun Sun, Mila Janjic Rankovic, Matthias Folwaczny, Thomas Stocker, Sven Otto, Andrea Wichelhaus, Uwe Baumert

**Affiliations:** 1 Department of Orthodontics and Dentofacial Orthopedics, University Hospital, LMU Munich, 80336 Munich, Germany; cysun.stomatology@gmail.com (C.S.); mila_janjic@yahoo.com (M.J.R.); th.stocker@med.uni-muenchen.de (T.S.); kfo.sekretariat@med.uni-muenchen.de (A.W.); 2 Department of Conservative Dentistry and Periodontology, University Hospital, LMU Munich, 80336 Munich, Germany; mfolwa@dent.med.uni-muenchen.de; 3 Department of Oral and Maxillofacial Surgery and Facial Plastic Surgery, University Hospital, LMU Munich, 80336 Munich, Germany; sven.otto@med.uni-muenchen.de

**Keywords:** periodontal ligament cells, tensile strain, bone remodeling, stretching, orthodontic tooth movement

## Abstract

This study aimed to investigate the effects of different magnitudes and durations of static tensile strain on human periodontal ligament cells (hPDLCs), focusing on osteogenesis, mechanosensing and inflammation. Static tensile strain magnitudes of 0%, 3%, 6%, 10%, 15% and 20% were applied to hPDLCs for 1, 2 and 3 days. Cell viability was confirmed via live/dead cell staining. Reference genes were tested by reverse transcription quantitative real-time polymerase chain reaction (RT-qPCR) and assessed. The expressions of *TNFRSF11B*, *ALPL*, *RUNX2*, *BGLAP*, *SP7*, *FOS*, *IL6*, *PTGS2*, *TNF*, IL1B, IL8, IL10 and PGE2 were analyzed by RT-qPCR and/or enzyme-linked immunosorbent assay (ELISA). *ALPL* and *RUNX2* both peaked after 1 day, reaching their maximum at 3%, whereas *BGLAP* peaked after 3 days with its maximum at 10%. *SP7* peaked after 1 day at 6%, 10% and 15%. *FOS* peaked after 3 days with its maximum at 3%, 6% and 15%. The expressions of *IL6* and *PTGS2* both peaked after 1 day, with their minimum at 10%. PGE2 peaked after 1 day (maximum at 20%). The ELISA of IL6 peaked after 3 days, with the minimum at 10%. In summary, the lower magnitudes promoted osteogenesis and caused less inflammation, while the higher magnitudes inhibited osteogenesis and enhanced inflammation. Among all magnitudes, 10% generally caused a lower level of inflammation with a higher level of osteogenesis.

## 1. Introduction

The aim of orthodontic tooth movement (OTM) is to align malpositioned teeth by applying external forces (“orthodontic forces”) to the teeth and thus stimulating bone remodeling [1]. Located between the teeth and the alveolar bone, the human periodontal ligament (hPDL) and the cells it contains play an essential role in withstanding mechanical forces in physiological, pathological and therapeutical conditions, e.g., orthodontic treatment [2].

During OTM, mechanical stimulation triggers complex aseptic inflammatory cellular and molecular processes causing the remodeling of the surrounding tissues, ultimately leading to bone resorption on the compression side and bone formation on the tension side. Inflammation is regulated by a large array of mediator molecules [3], including pro-inflammatory molecules such as interleukin 1B (IL1B), tumor necrosis factor (TNF), interleukin 6 (IL6) and interleukin 8 (IL8), as well as prostaglandin-endoperoxide synthase 2 (*PTGS2*; also known as *COX2*), prostaglandin E2 (PGE2) and anti-inflammatory molecules such as interleukin 10 (IL10) [4]. Various molecules mediating osteoclasto-/osteoblastogenesis are upregulated at different stages of bone remodeling during this aseptic inflammatory process [5], including transcription factors (e.g., *Runt*-related transcription factor 2 (*RUNX2*) and *SP7*, also known as osterix) [6,7,8] and early or late osteoblastic marker genes such as alkaline phosphatase (*ALPL*), bone-matrix protein-bone gamma-carboxyglutamate protein (*BGLAP*, also known as osteocalcin) [9], as well as receptor activator of nuclear factor kappa ligand (*RANKL*) and osteoprotegerin (*OPG*) [10]. The proto-oncogene FOS is an immediate/early gene essential for mechanical stimulation. Its dimerization with JUN forms the heterodimeric activator protein 1 (AP1), which then binds to different promoters of osteoblast-specific genes, activating the proliferation and differentiation of osteoblasts in periodontal tissue [2,11].

Appropriate mechanical loading is essential for the homeostasis and thus the controlled and coordinated remodeling of both the PDL and the alveolar bone. A lack of mechanical stimuli will lead to the atrophy of PDL and/or bone. In contrast, excessive force affects PDL and bone in a similar manner resulting in periodontal attachment loss and/or loss of alveolar bone, respectively, finally leading to uncontrolled tooth movement and/or root resorption [1,12]. Taken together, optimal therapeutical forces are crucial for well-regulated tissue remodeling. Clinically, therapeutic tooth movement is centrally based on careful mechanical stimulation as low and as short as possible, sufficient to achieve the desired biological responses [1,12]. Therefore, parameters of mechanical stimulation eligible to induce therapeutic tissue remodeling within the periodontal and osseous tissues need to be further defined [1,12].

To gain improved insight into the effects of therapeutic forces on the expression and regulation of relevant genes involved in tooth movement and bone remodeling, different in vitro force application models have been suggested specifically in terms of compressive forces [7,13,14,15]. Regarding tension, different in vitro models have been applied addressing distinct issues [16,17,18], i.e., apoptosis [19,20], pyroptosis [21], angiogenesis [22], osteogenesis [23] and inflammation [24], but most of these studies focused on specific tension magnitudes only. Even in those studies considering different magnitudes, tension was only applied for a single period of time [25,26,27,28].

Therefore, aim of this study was to investigate the effects of different tensile strain magnitudes and durations on human periodontal ligament cells (hPDLCs), with special emphasis on gene expression related to bone remodeling, mechanosensing and inflammation.

## 2. Results

**Figure 1:**
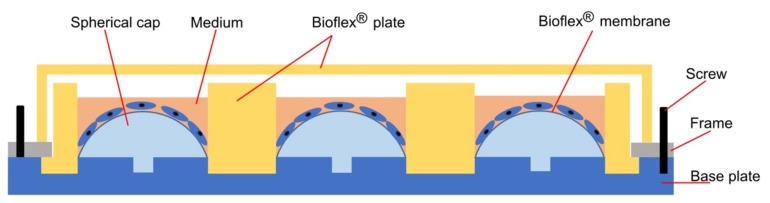
Experimental setup used to apply tensile strain: cells were seeded on the elastic silicone membrane of a BioFlex^®^ plate and incubated overnight. Afterward, spherical caps with defined shapes were inserted into the base plate to statically apply a 3%, 6%, 10%, 15% or 20% increase of the membrane area. No tensile strain was applied to the control wells. After placing the BioFlex^®^ plate onto the base plate, the outer frame was fixed with screws, thus applying tensile strain of predefined magnitudes to the cells growing on the membrane (more details can be found in Section 4.2).

A custom-made apparatus was constructed (Figure 1) to apply different magnitudes of static equibiaxial tensile strain to adherent cells growing on a flexible membrane. Static cell stretching was achieved by spherical caps placed below the membrane (Figure 1) leading to an increase in membrane area. Herein, static cell stretching of hPDLCs of different magnitudes (0% = control, 3%, 6%, 10%, 15 % and 20%) was applied to hPDLCs with the respective, matching spherical caps for 1, 2 and 3 days. In the remaining parts of this manuscript, the magnitude of static tensile strain was represented by the percentage of stretch applied. Cell viability was assessed by live/dead cell staining, and the expression of target genes related to bone remodeling, mechanosensation and inflammation was quantified using reverse transcription quantitative real-time polymerase chain reaction (RT-qPCR) and/or enzyme-linked immunosorbent assay (ELISA).

### 2.1. Cell Viability

**Figure 2:**
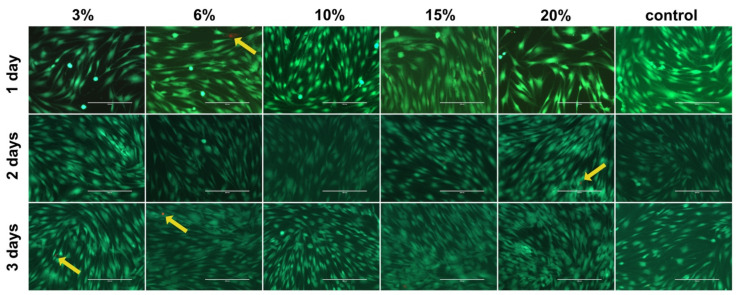
Cell viability of human periodontal ligament cells (hPDLCs) as assessed by live/dead cell staining. Microscopic images (bar: 200 μm) of cells growing in the center of each well were used herein, representing different tensile strain magnitudes and durations. Live cells are indicated by green staining, and dead cells are indicated by red staining (yellow arrows).

Since the maximum equibiaxial tensile strain is induced in the central part of the membrane [29], cells growing in this area of each membrane were used for viability testing with live/dead cell staining (Figure 2). The viability of hPDLCs remained unaffected as compared to the untreated control samples independent of tension magnitude and duration.

### 2.2. Reference Gene Selection

**Figure 3:**
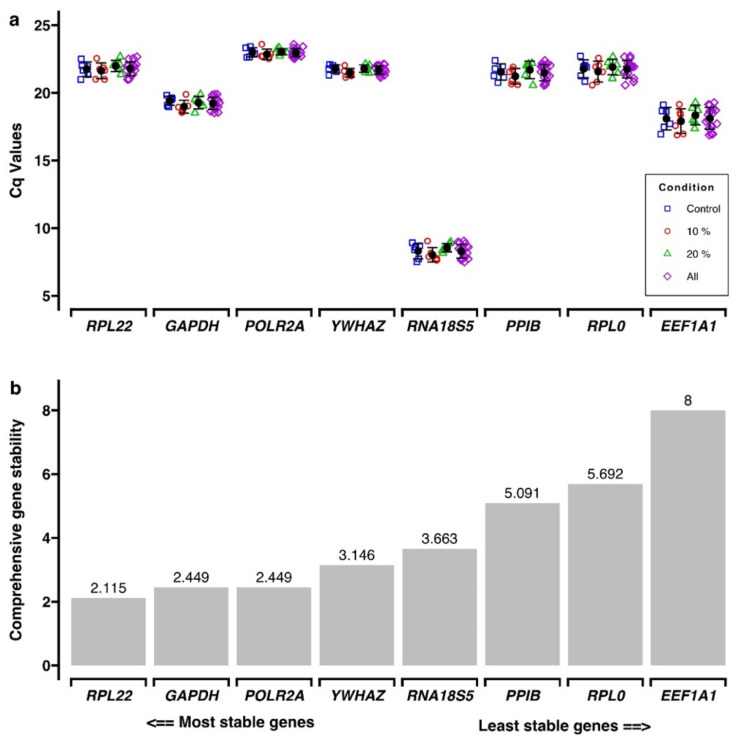
Reference gene primer stability was obtained with RefFinder. (**a**). C_q_ values for the panel of reference genes, using samples exposed to different tensile strain magnitudes for 1 and 3 days. Average expression (all) for the three magnitudes was also calculated. Six quantitative real-time polymerase chain reaction (qPCR) runs were analyzed representing three biological replicates with two technical replicates each (Supplement 1). (**b**). Comprehensive gene stability analysis for the panel of reference genes. Lower values indicate higher gene stability (Supplement 1).

For validation of reference genes, the expression of a panel of eight pre-selected genes was assessed with RT-qPCR using samples exposed to 0%, 10% and 20% cell stretching for 1 and 3 days. Considering the C_q_ values, the most abundant reference gene was *RNA18S5* with mean C_q_ values ranging from 8.03 ± 0.53 to 8.55 ± 0.32 (Figure 3a; Supplementary Table S1.1).

The RefFinder program was used to identify the most stable reference gene within the panel, which calculated comprehensive gene stability values for each gene based on four different algorithms (Figure 3b; Supplementary Tables S1.2 and S1.3). Accordingly, *RPL22* (RefFinder gene stability: 2.115), *GAPDH* and *POLR2A* (both 2.449) were the most stable reference genes tested (Figure 3b and Supplement 1). The “Minimum Information for Publication of Quantitative Real-Time PCR Experiment” (MIQE) guidelines recommend using more than one reference gene for normalization to improve the quality of data [30]. Albeit RefFinder calculated the same gene stability values for both *GAPDH* and *POLR2A*, the latter was selected together with *RPL22* as reference genes for normalization, due to a comparable level of expression as for most of the target genes analyzed in this study.

### 2.3. Expression of Target Genes

Cell stretching was applied to hPDLCs for up to three days using six different magnitudes (0%, 3%, 6%, 10%, 15% and 20%). Afterward, expression of target genes was analyzed with RT-qPCR (reference genes: *RPL22* and *POLR2A*) and/or ELISA. A total of thirteen different loci were included, representing three different functional groups: bone-remodeling-related genes, including *ALPL*, *RUNX2*, *BGLAP*, *SP7*, TNF and *TNFRSF11B*, the mechanosensation-related locus *FOS* and the inflammation-related loci, including IL6, PTGS2, PGE2, IL1B, IL8 and IL10. The differences in gene expression between the test groups and the corresponding controls were analyzed. Tensile strain duration dependency was determined for each magnitude separately, and magnitude dependency was assessed for each duration. If not otherwise stated, mean fold changes (FC) and adjusted *P* (*P*_adj._) values after Bonferroni correction for multiple testing are reported. Descriptive statistics for each analyte and tension/duration combination are summarized in Table 1.

The protein concentrations of TNF, IL1B, IL8 and IL10 in the supernatants were all below the detection limit and were therefore not further analyzed. The gene expression of tumor necrosis factor-alpha receptor superfamily member 11B (*TNFRSF11B*, also known as *OPG*) was below the detection limit (C_q_ values > 35) and therefore not further analyzed.

#### 2.3.1. Bone-Remodeling-Related Target Genes

**Figure 4:**
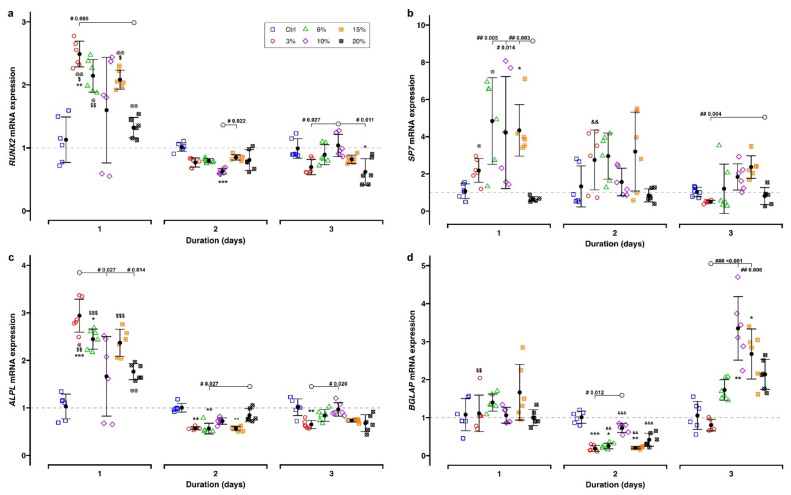
Reverse transcription quantitative real-time polymerase chain reaction (RT-qPCR) results for genes related to bone remodeling after 1 to 3 days of static tensile strain application: (**a**) *RUNX2*, (**b**) *SP7*, (**c**) *ALPL* and (**d**) *BGLAP*. Each experimental unit is summarized by mean (⚫) and error bars representing SD. The ΔΔCq method was applied, and *RPL22* and *POLR2A* were used as reference genes. Gene expression of controls is indicated by the gray dashed line. Analysis of differences between the test and control groups was carried out with the Kruskal–Wallis test followed by Bonferroni correction for multiple testing. Significant differences between groups are indicated as follows: *, test group vs. corresponding control; effects of duration: $, 1 day vs. 2 days; @, 1 day vs. 3 days; &, 2 days vs. 3 days; effects of magnitudes (“#”) are indicated by *P*_adj._ values; “◯” defines the counterpart of comparisons. Levels of significance: *P*_adj._ < 0.05: *, $, @, #; *P*_adj._ < 0.01: **, $$, @@, &&, ##; *P*_adj._ < 0.001: ***, $$$, &&&, ###.

Expression of bone-remodeling-related genes *ALPL*, *RUNX2*, *BGLAP* and *SP7* was evaluated using RT-qPCR (reference genes: *RPL22* and *POLR2A*). The results are shown in Figure 4 and summarized in Table 1.

Generally, the gene expression of *RUNX2* and *ALPL* showed similar dependency on the duration and magnitude of cell stretching. The transcription of both genes showed a significant increase after 1 day of cell stretching (*RUNX2* mean range: 1.32–2.49; *ALPL* mean range: 1.66–2.94), which declined at Days 2 and 3 and ultimately reached control levels (*RUNX2*: 0.62–1.04; *ALPL*: 0.56–0.97) (Figure 4a,c). For both genes, the highest gene expression was found after 3% cell stretching at Day 1 (*RUNX2*: 2.49 ± 0.20, *P_adj._* = 0.001; *ALPL*: 2.94 ± 0.35, *P_adj._* < 0.001), showing significant differences in comparison to further test groups (*RUNX2*: 3% vs. 20%, *P_adj._* = 0.005; *ALPL*: 3% vs. 10%, *P_adj._* = 0.027, 3% vs. 20%, *P_adj._* = 0.014). The transcriptional activity of both genes increased less after exposure to higher stretching levels (*RUNX2*: 20%, 1.32 ± 0.16; *ALPL:* 10%, 1.66 ± 0.84 and 20%, 1.76 ± 0.17), and differences compared to the controls did not reach statistical significance (*P_adj._* > 0.05). Independent of the stretching level, the expression of both target genes either returned to the control levels or was even lower at Days 2 and 3.

Identical to *RUNX2* and *ALPL*, *SP7* expression was also inversely related to the duration of the mechanical stimulation (Figure 4b). The specific amount of stretching applied to the cells caused inconsistent effects on *SP7* gene expression. While lower tensile strain levels (6%, 10% and 15%) led to significant upregulation, maximum cell stretching (20%) induced a downregulation of *SP7* expression which did not reach significance.

Except for 3% magnitude, the *BGLAP* gene was also differentially expressed, mostly depending on the duration of the mechanical stimulation (Figure 4d, Table 1). After 1 day, its gene expression was initially not statistically different from the corresponding controls (mean FC range: 1.00–1.67). A statistically significant downregulation was found for the remaining tensile strain magnitudes (mean FC range: 0.21–0.42) except 10% (mean FC: 0.73) after 2 days. After 3 days of 10% and 15% tensile strain application, a statistically significant upregulation of *BGLAP* was found (FC; 10%: 3.35 ± 0.84; 15%: 2.68 ± 0.66). Cell stretching of 3% led to a significant temporary downregulation (0.19 ± 0.08, *P_adj._* < 0.001) at Day 2 only. Maximum *BGLAP* gene expression was identified with 10% cell stretching at Day 3 (FC: 3.35 ± 0.84, *P_adj._* = 0.001).

#### 2.3.2. Mechanosensation-Related Target Genes

**Figure 5:**
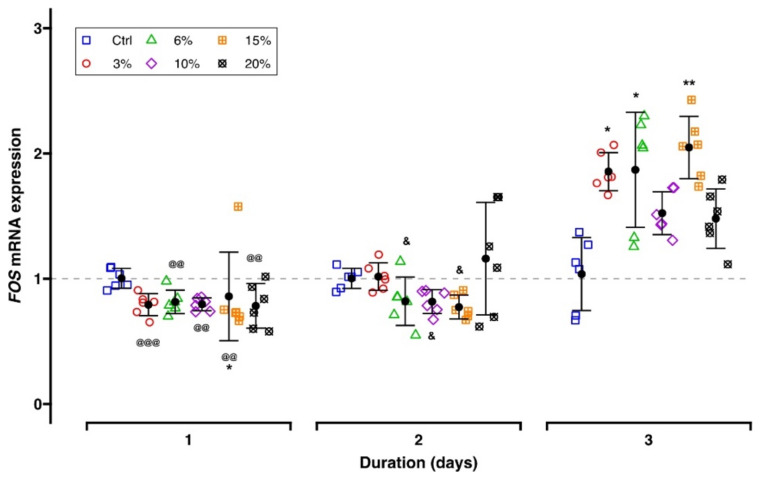
RT-qPCR results of the mechanosensation-related gene *FOS*. Each experimental unit is summarized by mean (⚫) and error bars representing SD. The ΔΔCq method was applied, and *RPL22* and *POLR2A* were used as reference genes. Gene expression of controls is indicated by the gray dashed line. Analysis of differences between the test and control groups was carried out with the Kruskal–Wallis test followed by Bonferroni correction for multiple testing. Significant differences between groups are indicated as follows: *, test group vs. corresponding control; effects of duration: @, Day 1 vs. Day 3; &, Day 2 vs. Day 3. Levels of significance: *P*_adj._ < 0.05: *, &; *P*_adj._ < 0.01: **, @@, *P*_adj._ < 0.001: @@@.

Generally, *FOS* gene expression remained unchanged at Days 1 and 2, independent of the tensile strain magnitude (mean FC range: 0.77–1.16; Figure 5, Table 1). After three days of cell stretching, upregulation was induced (mean FC range: 1.48–2.05) showing no differences between the various force magnitudes.

#### 2.3.3. Inflammation-Related Target Genes

**Figure 6:**
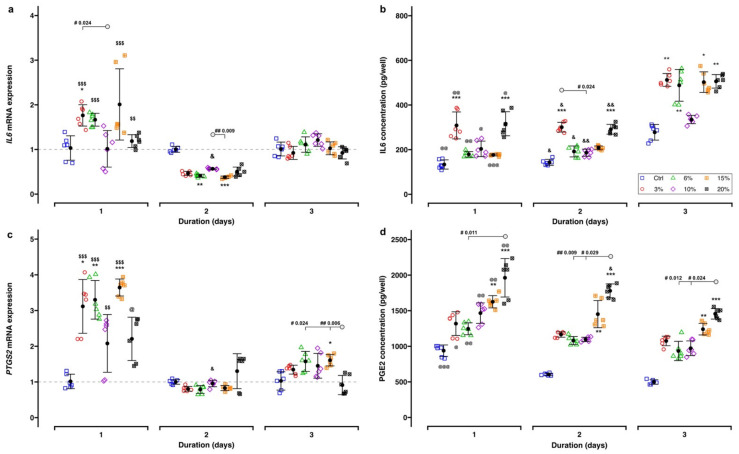
Expression of inflammation-related genes and metabolites: (**a**) *IL6* gene, (**b**) IL6 in the supernatant, (**c**) *PTGS2* gene, (**d**) PGE2 in the supernatant. Each experimental unit is summarized by mean (⚫) and error bars representing SD. The ΔΔCq method was applied, and *RPL22* and *POLR2A* were used as reference genes. Gene expression of controls is indicated by the gray dashed line. Analysis of differences between the test and control groups was carried out with the Kruskal–Wallis test followed by Bonferroni correction for multiple testing. Significant differences between groups are indicated as follows: *, test groups vs. corresponding control; effect of tensile strain duration: $, Day 1 vs. Day 2; @, Day 1 vs. Day 3; &, Day 2 vs. Day 3; effect of tensile strain magnitude (“#”) are reported with *P*_adj._ values and “◯” defines the counterpart of comparisons. Levels of significance: *P*_adj._ < 0.05: *, @, &, #; *P*_adj._ < 0.01: **, $$, @@, &&, ##; *P*_adj._ < 0.001: ***, $$$, @@@.

The gene expressions of *IL6* and *PTGS2* on the transcriptional level, as well as the corresponding ELISA results, are given in Figure 6 and Table 1.

Generally, *IL6* gene expression was increased in cells exposed to tensile strain as compared to the controls at Day 1 (mean FC range: 1.01–2.01), but it was significantly attenuated at Day 2 (mean FC range: 0.38–0.57), finally returning to control levels at Day 3 (mean FC range: 0.92–1.21) (Table 1, Figure 6a). The maximum *IL6* gene expression was observed at Day 1 following the application of 15% cell stretching (FC: 2.01 ± 0.80), whereas the lower magnitudes of 3% and 6% induced a less pronounced upregulation (mean FC range: 1.67–1.76). *IL6* gene expression remained unaffected for both the 10% and 20% tensile strain. Two days of cell stretching led to a downregulation of *IL6* gene expression (mean FC range: 0.38–0.57), which was statistically significant for the 6% (FC: 0.41 ± 0.03, *P_adj._* = 0.002) and 15% tensile strain (FC: 0.38 ± 0.03, *P_adj._* < 0.001) compared to the corresponding controls only. After 3 days of tensile strain application, *IL6* gene expression reached the level of the corresponding control.

Regarding the translational level, IL6 concentration in the cell culture supernatant was dependent on the duration of cell stretching (Table 1, Figure 6b). It was elevated during the first two days in comparison to untreated controls (176.3–316.1 pg/well, which corresponded to a ratio of 1.32–2.37) followed by a further increase after 3 days of cell stretching independent of its magnitude (335.0–512.4 pg/well; ratio: 1.21–1.85). The highest IL6 concentrations were identified with 3% (1 day: ratio = 2.31, *P_adj._* < 0.001; 2 days: 2.10, *P_adj._* < 0.001; 3 days: 1.85, *P_adj._* = 0.006) and 20% tensile strain independent of the duration (1 day: 2.37, *P_adj._* < 0.001; 2 days: 2.04, *P_adj._* < 0.001; 3 days: 1.82, *P_adj._* = 0.008). Generally, the lowest IL6 concentrations were found after 10% cell stretching at all time points.

*PTGS2* gene expression was initially upregulated (Day 1) (mean FC range: 2.08–3.64) (Table 1, Figure 6c), showing the strongest increase for the 15% tensile strain (FC: 3.64 ± 0.24, *P_adj._* < 0.001). At Day 2 (mean FC range: 0.79–1.30) *PTGS2* gene expression remained unchanged, whereas at Day 3 almost no effect on gene expression was observed (mean FC range: 0.91–1.61).

Exposure of hPDLCs to tensile strain caused a significant upregulation of PGE2 during the entire observation period, reaching the maximum amount at Day 1 (1246.0–1962.5 pg/well, which corresponded to a ratio of 1.33–2.09 relative to the control) (Table 1, Figure 6d). With lower levels of tensile strain (3%, 6% and 10%), no differences were found compared to the unexposed control in terms of PGE2 expression. Only magnitudes of 15% (1 day: 1.73, P*_adj._* = 0.001; 2 days: 2.40, P*_adj._* = 0.001; 3 days: 2.49, P*_adj._* = 0.001) and 20% (1 day: 2.09, P*_adj._* < 0.001; 2 days: 2.95, P*_adj._* < 0.001; 3 days: 2.92, P*_adj._* < 0.001) significantly amplified PGE2 concentrations.

## 3. Discussion

Physical loading induces both compression and tensile forces in tissues and cells, influencing and modulating their physiology. It is well established that tension triggers cellular mechanisms, including those leading to bone remodeling at the whole-body level. As such, physiological activities in the orofacial region such as mastication and various non-physiological impacts, i.e., therapeutical stimulation during orthodontic tooth movement induce tensile forces in the periodontal ligament (PDL), promoting tissue and especially bone remodeling therein.

Therefore, the effect of tension on hPDLCs has been widely addressed in numerous in vitro studies, which have been recently reviewed [16,31]. In most of these in vitro studies, the effects of exclusively one specific tensile strain magnitude were analyzed, commonly for a maximum of 48 h using tension devices based on cells growing on a flexible membrane, similar to the one proposed in this study [16]. However, the combined effects of different magnitudes together with various durations on hPDLCs have rarely been compared. Herein, we applied different levels of cell stretching (i.e., 3%, 6%, 10%, 15% and 20%) for various periods of time (i.e., 1, 2 and 3 days), to cover a broad range of tensile strain parameters, and analyzed their impact on the regulation of bone remodeling, mechanosensing and inflammation processes in hPDLCs.

### 3.1. Selection of Tensile Strain Parameters

In a recently published systematic review [16], different sources were identified to deduce clinical relevant tensile strain magnitudes for in vitro studies: (1) tensile strain derived from mastication or OTM [32,33,34,35], (2) finite element simulation including biomechanical confirmation [36,37], (3) the specific anatomy of the periodontium including the PDL width [38], or (4) previously published studies [39,40,41]. Based on finite element simulations and biomechanical testing, it was shown that bodily movement of a premolar tooth with 1 N pressure induced 1% strain in the PDL on the tension side [36], whereas strains of 6–7% for intrusive and 8–25% for horizontal tooth movement in the PDL were reported after application of 3 N intrusive loading [37]. Similar results were obtained considering the specific anatomy of the PDL in vivo: application of 1 N and 3 N forces to incisors led to an increase in PDL width of ~12% [38]. A recent systematic review reported 10% tensile strain as the most frequently applied magnitude [16]. Herein, 10% tensile strain was selected as being equivalent to a physiological force exposure, whereas 20% cell stretching is commonly used to investigate the influence of pathogenic stimuli on cellular mechanotransduction in hPDLCs [39,40,41] and therefore was considered the upper limit of tensile strain in this study. Magnitudes of 3% and 6% were applied to determine the lower limit of the mechanical stimulus affecting gene expression [39,41].

In line with most of the previous studies, the total observation period in the current study did not exceed 3 days, avoiding the repetition of cell feeding, which might have unpredictably affected cellular physiology. Based on these considerations, tensile strain magnitudes of 0%, 3%, 6%, 10%, 15% and 20% were applied for 1, 2 and 3 days in this study.

### 3.2. Effect of Different Parameters of Tensile Strain on Bone Remodeling

The osteogenic differentiation of hPDLCs plays an essential role in the bone remodeling of the periodontium [9,42]. Different genetic loci are involved in its regulation, including but not limited to *RUNX2*, *SP7*, *ALPL*, *BGLAP* and *TNFRSF11B*. Among them, *RUNX2* is considered as one of the key regulators of bone remodeling that is upregulated in both preosteoblasts and immature osteoblasts but downregulated in mature osteoblasts [43]. Expression of *RUNX2* is stimulated by mechanical compression in osteoblasts [13] and mechanical tension in hPDLCs [44]. *RUNX2* is essential for the regulation of several downstream loci involved in osteoblast differentiation and bone-matrix synthesis, including alkaline phosphatase (*ALPL*) and osterix (*SP7*) [45,46]. Being an essential transcription factor for osteogenic differentiation the latter acts downstream of *RUNX2* [6,47]. Belonging to the zinc finger-containing transcription factors of the SP family, *SP7* is expressed in osteoblasts. Its expression is essential for the differentiation of preosteoblasts into mature osteoblasts [6,45]. ALPL expression has been commonly accepted as an early marker for new bone formation [48], which in turn is activated by *RUNX2*, and thus is essential for osteoblast maturation [9,48]. It plays a central role in osteogenic mineralization, bone calcification and mineralization [49,50,51]. *BGLAP* is the most abundant non-collagenous bone-matrix protein produced by mature osteoblasts [49]. Its expression is regulated among others by *RUNX2* and *SP7* [9,45], and it is highly expressed in the late stage of osteoblast differentiation and mineralization [49].

In this study, expression of *RUNX2*, *SP7*, *ALPL* and *BGLAP* roughly followed a time dependent pattern, showing similarity with the timeline of events occurring during osteogenesis [9]. *RUNX2*, as one of the key regulators of bone remodeling, was upregulated after 1 day of cell stretching only. This finding is consistent with a previous study, showing a considerably amplified *RUNX2* expression within the first 24 h of cell stretching, increasing 6 h after the start of the mechanical stimulation [52]. *RUNX2* upregulation has also been observed in several studies applying dynamic tensile strain [23,42,48,53].

Comparable with the *RUNX2* gene, the *ALPL* gene was also differentially expressed depending on the observation period. The maximum upregulation of *ALPL* gene expression was identified after 1 day of tensile strain application, followed by a slight downregulation afterward, with lower levels (3% and 6%) resulting in stronger gene expression. Similar findings have been reported for 1% and 5% tensile strain [54], whereas other studies report no correlation between *ALPL* gene expression and tensile strain magnitude [49,55]. These contradicting results might be due to differences in the experimental conditions.

*SP7*, another gene acting downstream of *RUNX2* in the osteoblast differentiation pathway, showed a sustained upregulation, which was most evident for 6% to 15% tensile strain, while 20% caused an adverse effect independent of its duration. The maximum upregulation of *SP7* gene expression was identified at Day 1 for most magnitudes. These results were consistent with other studies, which reported upregulation of *SP7* in hPDLCs after 1 day of tensile strain application [23,45,47]. Similar to *RUNX2*, downregulation was found herein for 20% magnitude at all time points, indicating an inhibitory effect of high magnitude cell stretching for osteogenic differentiation.

*BGLAP* expression tended to increase with the length of cell stretching applied, revealing the highest expression after 3 days at 6% to 20%. A similar late regulation pattern of *BGLAP* has been reported in other studies [49,54]. The decreasing expression of *BGLAP* after 2 days of cell stretching observed herein might be explained by the cell proliferation of young osteoblasts, considering the heterogeneous characteristics of hPDLCs [56] and the specific characteristic of *BGLAP*, which is mainly upregulated in mature osteoblasts [9]. Similarly, downregulation of *BGLAP* was also identified in stretched osteoblasts, which was attributed to the presence of osteoblasts in different stages of development [54].

Interestingly, *TNFRSF11B* gene expression was below the detection limit in all experimental conditions in this study. In contrast, upregulation of *TNFRSF11B* has been reported previously in hPDLCs after exposure to static tensile strain for 12 h but not for longer periods of time [16,54,57]. Thus, one might assume that the *TNFRSF11B* gene is already upregulated by tensile strain in shorter time periods than covered in the present study.

### 3.3. Effect of Different Parameters of Tensile Strain on Mechanosensing

*FOS* is an immediate/early response gene which is essential for the perception of mechanical stimulation. Dimerization of FOS with JUN creates the active heterodimeric transcription factor AP1, which plays a central role in osteoblast proliferation and differentiation [11,34]. Regardless of tension magnitudes, upregulation of *FOS* in hPDLCs was only observed after 3 days of tension in this study. Other studies have reported upregulation of *FOS* in stretched hPDLCs as early as after 15 min [58,59] and 3 h [60] of tensile strain application. Shorter force application intervals should be added in further studies, considering the early response characteristics of *FOS*.

### 3.4. Effect of Different Parameters of Tensile Strain on Inflammation

It has been demonstrated that inflammation is induced by mechanical stimuli during OTM [3] and regulated by several cytokines and chemokines [61]. Among others, IL6, PGE2, *PTGS2*, TNF, IL8 and IL1B play key roles in inflammation and bone resorption, while IL10 is regarded as an anti-inflammatory mediator. As an inflammatory cytokine, IL6 is involved in osteoclastogenesis [4,52] and its regulation by tensile strain has been reported in several previous in vitro studies [52,55,62,63].

In this study, *IL6* gene expression was upregulated after 1 day of cell stretching depending on the magnitude, but no effect was found after 3 days. In contrast, on the translational level the total amount of IL6 increased with the length of cell stretching for all magnitudes in the current study. In a study of Jacobs et al. [62] no significant time dependency was found for *IL6* gene expression, but it tended to correlate with the strength of tensile strain application. It was suggested that lower strain magnitudes induce anti-inflammatory effects whereas higher magnitudes lead to pro-inflammatory effects [62]. A similar conclusion was proposed by Wada et al. [52], who reported an upregulated *IL6* gene expression after 15% strain application within the first 24 h. On the other hand, Nazet et al. [55] reported a decreased *IL6* gene expression after 16% and 35% tensile strain applied for 48 h. The inconsistent results observed in these studies might be due to the pro- and anti-inflammatory properties of IL6, together with the complex regulation pathway [63].

*PTGS2* is a key enzyme involved in PGE2 biosynthesis and both play essential roles in inflammation and bone resorption in response to mechanical stimuli [64,65]. Generally, increasing *PTGS2* expression and PGE2 synthesis is reported after exposure of hPDLCs to increasing magnitudes of static tensile strain [55,62]. Concerning the effects of tensile strain duration on *PTGS2* gene expression, partially contradicting results have been reported [52,55]. Interestingly, no significant difference in the transcription of the *PTGS2* gene for different magnitudes was found herein in general, whereas PGE2 expression was significantly amplified after tensile strain application with higher magnitudes (15% and 20%). Taken together, it seems reasonable to assume that higher magnitudes of tensile strain lead to increased inflammation and should thus be avoided in clinical situations.

IL1B, TNF, IL8 and IL10 were below the detection limits of the ELISA systems applied in this study. So far, expression of IL8 and IL10 after tensile strain application has been reported for cells exposed to dynamic tensile strain only [4,66]. Though upregulation of IL1B and TNF has been previously reported for static tensile strain [49,52], the different molecular response might be due to individual difference among cells from different donors.

A growing portion of orthodontic patients presents with periodontal disease [1,67,68]. Periodontitis has been associated not only with numerous chronic systemic inflammatory and autoimmune conditions, such as atherosclerosis, diabetes mellitus and rheumatoid arthritis [69], but also with macular degeneration [70] and colorectal cancer [71]. It has been hypothesized that pro-inflammatory mediators (e.g., IL6 and IL1B), which are highly expressed locally within inflamed periodontal tissue, will be spread systematically, ultimately amplifying different pre-existing non-oral inflammation [71]. It has been proposed that OTM and periodontitis share some molecular pathways, particularly in terms of inflammation and osteogenesis/osteclastogenesis. Yet, the interrelation between both conditions as well as the association between periodontitis and several distinct systemic diseases remain to be elucidated. Herein, the expression of several genes coding for pro-inflammatory mediators was positively correlated with the exposure of PDL cells against tensile strain. In order to avoid an exaggerated expression of pro-inflammatory stimuli during orthodontic tooth movement that might additionally enhance periodontitis-associated inflammation and tissue destruction, lower therapeutic forces might be appropriate for the orthodontic treatment of patients with a history of periodontitis as compared to periodontally healthy patients.

### 3.5. Clinical Relevance

In this study the effects of different tension parameters applied to hPDLCs were analyzed with reference to bone remodeling, mechanosensing and inflammation. The expression of genes regulating bone remodeling was clearly dependent on the duration of tensile strain application. Generally, lower magnitudes (≤15%) enhanced bone remodeling, whereas 20% tensile strain attenuated bone remodeling. Upregulation of inflammation-related genes was correlated with higher tensile strain (15% and 20%). Though it is difficult to transfer in vitro results to the in vivo situation, it seems to be reasonable to assume that excessive forces might lead to adverse effects in clinical situations, especially in patients with a high susceptibility of periodontitis or associated diseases. Light orthodontic forces seem to be beneficial for coordinated bone remodeling and the maintenance of periodontal tissue homeostasis, ultimately enabling efficient tooth movement.

### 3.6. Strengths and Limitations of the Study

In this study, an apparatus was designed and manufactured to apply different levels of tensile strain simultaneously. This was achieved by using 3D designed and printed caps, which allowed the parameterized variation of tensile strain magnitudes.

Different tensile strain magnitudes and durations were applied to hPDLCs derived from the same donor and treated the same way throughout all experimental procedures. Regulation of genetic loci related to bone remodeling, mechanosensation and inflammation were investigated in this study, which was followed by a comprehensive analysis of their strain magnitude and duration-related expression.

Additionally, the stability of a panel of reference genes was evaluated using samples from the experimental condition and the corresponding controls and analyzed by four different algorithms calculating reference gene stability. Based on the computational analysis, the two most stable reference genes were selected to reduce variations in RT-qPCR experiments [30].

Though a comprehensive range of tensile strain magnitudes and durations was selected in this study, the first sampling took place after 1 day of strain application. Thus, early response genes, such as *FOS*, might have been undetectable. Taking this into account, additional earlier sampling points with regard to the initiation of the force application might have been appropriate.

Sample collection was performed after unloading of the experimental setup. Its disassembly and the time needed for sample preparation comprised a relevant delay between unloading and sample collection (i.e., cell lysates for gene expression studies, or collection of cell culture supernatants), leading to a possible change of cellular activity and/or physiology.

The PDL is considered as the main target of mechanical stimulation within the periodontium [12,72]. The effects of in vitro mechanical stimulation on PDL cells have been summarized in several recent reviews [16,17,18,73,74]. Independent of the specific isolation technique applied, it is commonly accepted that isolated PDL cells in fact represent a heterogeneous cell phenotype, which is primarily determined by the anatomical origin (middle third of the root) and the fibroblastic growth characteristics [56,75]. Long-term cultivation of hPDLCs typically leads to changes in cell morphology, growth rate, gene expression and response to mechanical stimulation [16,56,76,77,78]. To address this problem and to increase phenotypic homogeneity, hPDLCs are normally used in in vitro studies only with low passage numbers (passage ≤ 7) [56]. Nevertheless, it should be mentioned that clonal selection has been observed in hPDLCs as early as Passage 2, whereas *RUNX2*, *COL1A1* and *ALPL* expressions were not shown to be affected by passage number [79]. Yet, the particular phenotype of PDL cells as used herein has not been additionally confirmed based on molecular markers. Only a comparably small number of primary PDL cells can be harvested from one healthy donor. Hence, a balance between absolute cell amount and passage number must be achieved to conduct cell culture experiments, if pooling of cells from different individuals or the usage of immortalized primary cell lines should be avoided [56,80]. Since many different parameters have been tested herein to thoroughly delineate the effects of tensile strain on hPDLCs, a considerably large number of donor-specific cells was required. Thus, hPDLCs isolated according to standard protocols originating from one donor were used at Passages 5–6 [56].

Nevertheless, to gain insight into the biological variability of gene regulation during tensile strain application, cells derived from different donors should be included in future studies [60,81].

## 4. Materials and Methods

### 4.1. Primary Cell Culture

HPDLCs were obtained from the healthy first premolars of a 15-year-old female, which were removed due to orthodontic reasons with informed consent from the patient and her legal custodian. This study was conducted in accordance with the Declaration of Helsinki (https://www.wma.net/what-we-do/medical-ethics/declaration-of-helsinki/; accessed on 27 January 2022). Approval for the collection and use of hPDLCs was obtained from the ethics committee of the Ludwig-Maximilians-Universität München (project number 045-09).

Cells were derived from tissue samples obtained from the middle third of the roots using the explant technique as described by Somerman, et al. [75]. HPDLCs were cultivated with low glucose DMEM (21885025, Gibco, Life Technologies, Carlsbad, CA, USA) supplemented with 10% FBS (F7524; Sigma-Aldrich, St. Louis, MO, USA), 2% MEM vitamins (M6895; Biochrom, Berlin, Germany) and 1% of antibiotic/antimycotic (15240-062; Life Technologies, Carlsbad, CA, USA). Cells were grown in a humidified atmosphere with 5% CO_2_ at 37 °C and passaged in regular intervals of 3 to 4 days using 0.05% trypsin-EDTA solution (59417C; Sigma-Aldrich, St. Louis, MO, USA). Cells from Passages 5–6 were used in all experiments.

### 4.2. Tensile Strain Application Using a Custom-Made Tension Apparatus

**Figure 7:**
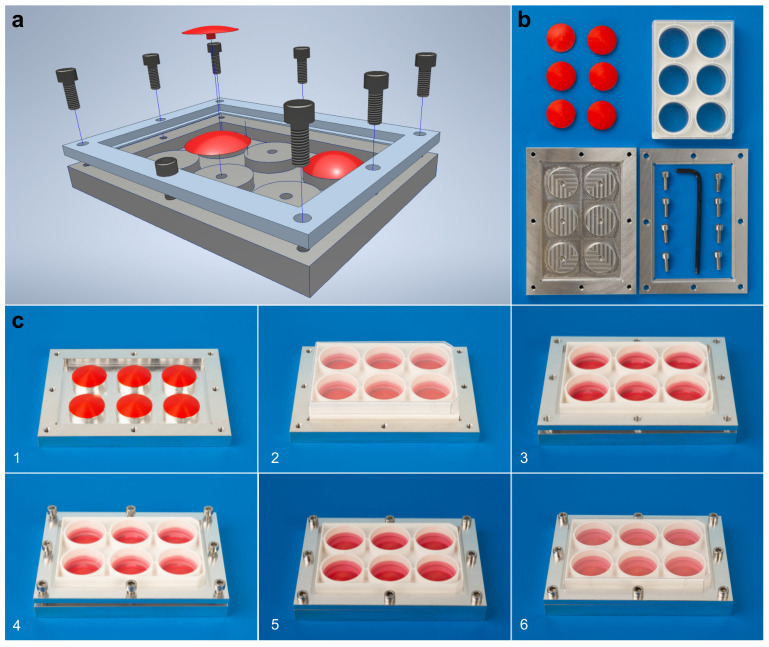
Experimental setup used to apply tensile strain. The apparatus consisted of 5 parts: the base plate, the pinned spherical cap, the BioFlex^®^ plate, the frame and the screws. After assembly, the cell-attached membrane was fitted onto the pinned spherical cap and stretched, producing predefined magnitudes of tensile strain. (**a**) 3D representation of the apparatus; (**b**) parts of the apparatus; (**c**) step-by-step (1–6) assembly of the apparatus.

Based on previous publications [55,82], an apparatus (Figure 1 and Figure 7) was constructed: (1) to apply different magnitudes of static equibiaxial tensile strain to adherent cells and (2) to feed cells without the relaxation of the flexible substrate delivering tensile strain to these cells. The apparatus consisted of five parts: a “base plate”, 3D-printed pinned spherical caps, a BioFlex^®^ Collagen-I coated Culture Plate (BF-3001C, Flexcell Intl. Corp., Hillsborough, NC, USA), a “frame” and screws (Figure 7).

The base plate, frame and spherical caps were constructed using a CAD program (Autodesk^®^ Inventor^®^ Professional 2019, Autodesk Inc., San Rafael, CA, USA) according to data published elsewhere [55,82]. Prototypes of the base plate, the frame and spherical caps representing different tensile strain levels [55] (Table 2) were printed with a 3D printer (Ultimaker 3, Ultimaker, Geldermalsen, The Netherlands) using a polylactic acid filament (Ultimaker PLA filament 2.85 mm, part no. 1618, Ultimaker); a polyvinyl alcohol filament was used to print the support structures (Ultimaker PVA filament 2.85 mm, Ultimaker). The final base plates and frames were made from aluminum by the workshop of the Department of Physics, LMU München. The 3D-printed spherical caps were used in the experiments.

HPDLCs were seeded at a cell density of 1.2 × 10^5^ cells/well on 6-well collagen-I coated BioFlex^®^ Culture Plates (Flexcell Intl. Corp., Hillsborough, NC, USA) and incubated overnight. Immediately prior to the assembly of the apparatus, the culture medium was changed. Then, the tensile strain application apparatus was assembled in a sterile environment as follows (Figure 7b,c): (1) 3D-printed spherical pinned caps were symmetrically inserted into the aluminum base plate; (2) the cover of the BioFlex^®^ plate was removed, and the plate was vertically placed onto the caps; (3) the frame was placed on the edges of the plate and screws were crosswise tightened symmetrically to fix the frame, plate and base plate and thus applying tensile strain to the adherent cells. The plate was covered and placed back into the CO_2_ incubator.

The elastic membranes including the attached cells were stretched by caps of different parameters (Table 2), resulting in an increase of membrane area of 0% (i.e., control), 3%, 6%, 10%, 15% and 20% and thus applying tensile strain to the cells [55]. Controls (0% tensile strain) were defined as wells without caps. All strain magnitudes were applied for 1, 2 and 3 days in two identical sets of apparatuses: one set for cell viability testing and the other for gene expression measurement and ELISA. For each tensile strain magnitude, three biological replicates were allocated for each duration. All assembled apparatuses were incubated and treated under the same conditions.

### 4.3. Cell Viability

The cell viability of hPDLCs for all magnitude/duration combinations was assessed using a live/dead cell staining kit (PK-CA707-30002, PromoKine, Heidelberg, Germany) according to the manufacturer’s instructions. Following strain release and removal of the supernatants, all wells were washed twice with PBS. Afterward, the BioFlex^®^ plates’ silicone membranes were cut out and placed in pre-labeled cell culture dishes (628160, CELLSTAR^®^, Greiner Bio-One GmbH, Frickenhausen, Germany). The membranes were then covered with fresh staining solution and incubated for 40 min in complete darkness at room temperature. Fluorescence microphotographs were taken of the centers of each membrane with a fluorescence microscope (EVOS^®^ *FL*, Invitrogen, Carlsbad, CA, USA) using 10× and 20× objectives.

### 4.4. Sample Preparation

After 1, 2 and 3 days of tensile strain application, the plate covers of the specific setup were removed. Cell culture supernatants from all wells were collected individually for ELISA (see below). The volume of each sample was recorded, and the samples were stored at −20 °C for further ELISA analysis. Next, the adherent cells were washed twice with sterile PBS, and cell lysates were prepared from each well using 750 µL RNA lysis buffer (R0160-1-50; Zymo, Irvine, CA, USA) according to the manufacturer’s instructions. Cell lysates were stored at −80 °C until all samples were collected.

### 4.5. Gene Expression Analysis

Analysis of *PTGS2*, *IL6*, *FOS*, *RUNX2*, *SP7*, *ALPL*, *BGLAP*, *TNFRSF11B* and *TNF* gene expressions following tensile strain application was carried out for all experimental magnitude/duration combinations according to previously described protocols [14]. A synopsis of the sample preparation and quantitative RT-PCR (RT-qPCR) is given below. A checklist according to the “Minimum Information for Publication of Quantitative Real-Time PCR Experiment” (MIQE) guidelines [30,83] is provided in Supplementary Table S2.1.

Total RNA preparation: Total RNA preparation was carried out using the Quick-RNA™ MicroPrep Kit (R1051; Zymo, Ivine, CA, USA) according to the manufacturer’s instructions. The following two steps were included in the procedure to reduce possible genomic DNA contamination: (I) defrosted cell lysates were centrifuged through QIAshredder™ columns (Qiagen, Hilden, Germany) before column purification; (II) during column purification, Dnase I digestion (Zymo) was applied. Finally, total RNA was eluted with 15 µL of Dnase/Rnase-free water (Zymo). Rnase inhibitor (Rnasin^®^, N2515; Promega, Madison, WI, USA) was added to each eluate at a final concentration of 1 U/µL. The total RNA preparations were stored at −80 °C until further use.

PCR primer selection: Generally, primer sequences were selected from public sources for both genes of interest and potential reference genes (Table 3). All primer pairs used were tested in silico according to the MIQE guidelines [30] as previously published [14] (Supplementary Tables S2.1 and S2.2). If not otherwise mentioned, unmodified primers were synthesized by Metabion GmbH (Planegg/Steinkirchen, Germany; Oligonucleotide Purification Cartridge OPC^®^ purification). Optimal annealing temperatures were determined with gradient PCR using the qPCR cycling program as specified in the MIQE checklist (Supplementary Table S2.1). Primer specificity was confirmed by agarose gel electrophoresis. Primer efficiencies were evaluated using standard curves prepared from serial dilutions of cDNA as specified in Supplementary Table S2.3 and quantified in the LightCycler^®^ 480 using the primer pairs detailed in Table 3.

Reference gene selection: A panel of reference genes (*EEF1A1*, *GAPDH*, *POLR2A*, *PPIB*, *RNA18SN5*, *RPL0*, *RPL22* and *YWHAZ*) was selected from public sources [55,84]. Evaluation of these reference genes was carried out using cDNA sampled from control, 10% and 20% after 1 and 3 days of static tensile strain application. RT-qPCR was performed as described below using gene-specific primers (Supplementary Table S2.3). The raw C_q_ values (Supplementary Table S1.1) were analyzed using RefFinder [85] (URL: https://www.heartcure.com.au/reffinder/ (accessed on 30 March 2021)), and the most stable genes were used as reference genes in RT-qPCR (Table 3).

RT-qPCR: Total RNA samples were thawed, and RNA concentration was determined photometrically (NanoDrop ND-1000, Peqlab, Erlangen, Germany). All RNA samples (600 ng each) were reverse transcribed to cDNA in a total reaction volume of 20 µL using the SuperScript™ IV First-Strand Synthesis System (18091050, Thermo Fisher Scientific, Waltham, MA, USA) and random primers as described by the manufacturer. Quantitative PCR was performed with the Luminaris Color HiGreen qPCR Master Mix Kit (K0392; Thermo Fisher Scientific, Waltham, MA, USA) following the instructions of the manufacturer using 2 µL cDNA (1:5 prediluted) in each PCR reaction. Each qPCR reaction included an initial Uracil-DNA glycosylase pre-treatment step to prevent carry-over contamination. Further details of the RT-qPCR reaction conditions are summarized in the MIQE checklist (Supplementary Table S2.1).

Gene expression calculation: Expression of target genes was quantified using the ΔΔCq method [86] with the selected reference genes *POLR2A* and *RPL22*. For each tension/duration combination, six qPCR reactions were analyzed representing three biological replicates with two technical replicates each.

### 4.6. Enzyme-Linked Immunosorbent Assay

Complete cell culture supernatant from all wells was collected for ELISA. The protein concentration of IL6, IL1B, IL8, TNF and IL10 was determined using the following DuoSet human ELISA kits (all from R&D Systems, Minneapolis, MN, USA): IL6 (DY206-05), IL1B (DY201-05), IL8/CXCL8 (DY208-05), TNF (DY210-5) and IL10 (DY217B-05). The PGE2 concentration in the cell culture supernatant was determined using the “PGE2 High Sensitivity ELISA kit” (ADI-931-001; Enzo Life Sciences (ELS) AG, Lausen, CH). All measurements were conducted using a microplate reader (Varioscan, Thermo Electron Corporation, Vantaa, Finland). For each magnitude/duration combination three biological replicates were measured twice. The measurements were reported as “concentration per well” (ng/well) using the well-specific volumes of each supernatant.

### 4.7. Statistics

Descriptive statistics of the gene expression and ELISA results are reported as mean ± standard deviation (SD) and 95% confidence intervals. All calculations were based on three biological replicates with two technical replicates for each gene/magnitude/duration combination. For each gene locus and marker molecule, differences between the different tensile strain magnitudes and durations were evaluated using the Kruskal–Wallis test followed by Bonferroni correction for multiple comparisons (*P*_adj._). All statistical procedures were carried out using IBM SPSS Statistics 27 (IBM Corp., Armonk, NY, USA). All test procedures were two-tailed considering *P*_adj._ values < 0.05 significant.

## 5. Conclusions

This study covered a broad range of tensile strain magnitudes and durations, with focus on bone remodeling, mechanosensing and inflammation. Generally, lower magnitudes were in favor of osteogenesis and resulted in less or even inhibited inflammation. Higher magnitudes led to an inhibition of osteogenesis and induced a higher level of inflammation. Among all magnitudes applied, 10% was optimal with higher levels of osteogenesis without evoking significant inflammation at the same time. The results showed an improved insight into the biological regulations of hPDLCs after exposure to different levels of tensile strain for a maximum period of 3 days. The current data might be useful in defining appropriate forces for OTM in clinical situations. Light orthodontic forces seem to be beneficial for coordinated bone remodeling and the maintenance of periodontal tissue homeostasis, ultimately enabling efficient tooth movement. The current results suggest that different force magnitudes might affect the expression of inflammatory- and bone-remodeling-related factors differently. These observations might be of relevance for future clinical studies, especially on interdisciplinary topics such as the application of orthodontic force as a regenerative stimulus to enhance periodontal defect healing.

## Figures and Tables

**Table 1:** Summary statistics and comparison of the effects of static tensile strain on target gene expression (*ALPL*, *BGLAP*, *PTGS2*, *FOS*, *IL6*, *RUNX2* and *SP7*) reported as fold change, PGE2 and IL6 in hPDLCs. Results are shown as mean (±SD) and 95% confidence interval [95% CI]. *P*-values were obtained with Kruskal–Wallis test (KW) and adjusted by the Bonferroni correction for multiple tests (adjusted *P*, *P*_adj._).

Analyte	Magnitude	Duration of Tensile Strain Application (Days)							KW of Duration (Magnitude Fixed)
		1 Day		2 Days		3 Days			
		Mean (SD)	[95% CI]	Mean (SD)	[95% CI]	Mean (SD)	[95% CI]	*P*_adj._	Sign.
*ALPL* (FC)	3	2.94 (0.35)	[3.35;3.37]	0.57 (0.03)	[0.57;0.63]	0.65 (0.09)	[0.70;0.80]	0.002	**
	6	2.45 (0.21)	[2.61;2.68]	0.56 (0.11)	[0.56;0.79]	0.84 (0.12)	[0.91;1.02]	0.001	**
	10	1.66 (0.84)	[2.45;2.52]	0.72 (0.07)	[0.75;0.82]	0.97 (0.14)	[1.08;1.20]	0.051	n.s.
	15	2.37 (0.28)	[2.58;2.76]	0.57 (0.05)	[0.58;0.62]	0.73 (0.03)	[0.76;0.76]	0.001	**
	20	1.76 (0.17)	[1.90;1.97]	0.84 (0.14)	[0.99;1.05]	0.68 (0.18)	[0.83;0.92]	0.002	**
KW of magnitude (duration fixed)		<0.001 ***		<0.001 ***		0.001 **			
*BGLAP* (FC)	3	1.11 (0.48)	[1.05;2.04]	0.19 (0.08)	[0.28;0.30]	0.80 (0.15)	[0.96;1.01]	0.001	**
	6	1.40 (0.23)	[1.62;1.70]	0.25 (0.07)	[0.32;0.35]	1.73 (0.27)	[2.05;2.08]	0.002	**
	10	1.06 (0.21)	[1.21;1.34]	0.73 (0.12)	[0.81;0.85]	3.35 (0.84)	[3.74;4.70]	0.001	**
	15	1.67 (0.73)	[2.25;2.85]	0.21 (0.03)	[0.22;0.24]	2.68 (0.66)	[3.04;3.40]	0.001	**
	20	1.00 (0.21)	[1.13;1.34]	0.42 (0.17)	[0.59;0.65]	2.14 (0.40)	[2.51;2.65]	0.001	**
KW of magnitude (duration fixed)		0.077 n.s.		<0.001 ***		<0.001 ***			
*PTGS2* (FC)	3	3.12 (0.75)	[3.47;4.07]	0.81 (0.07)	[0.83;0.93]	1.35 (0.12)	[1.45;1.48]	0.001	**
	6	3.30 (0.54)	[3.93;4.01]	0.79 (0.11)	[0.86;0.91]	1.58 (0.28)	[1.77;1.97]	0.001	**
	10	2.08 (0.81)	[2.63;2.72]	0.96 (0.09)	[1.02;1.05]	1.45 (0.35)	[1.82;1.95]	0.004	**
	15	3.64 (0.24)	[3.78;3.94]	0.83 (0.07)	[0.85;0.94]	1.61 (0.16)	[1.75;1.80]	0.001	**
	20	2.21 (0.61)	[2.74;2.76]	1.30 (0.49)	[1.65;1.65]	0.91 (0.27)	[1.22;1.26]	0.014	*
KW of magnitude (duration fixed)		<0.001 ***		0.017 *		0.001 **			
*FOS* (FC)	3	0.79 (0.09)	[0.83;0.91]	1.02 (0.11)	[1.08;1.19]	1.85 (0.15)	[2.01;2.07]	0.001	**
	6	0.81 (0.09)	[0.84;0.98]	0.82 (0.19)	[0.85;1.14]	1.87 (0.46)	[2.23;2.30]	0.003	**
	10	0.80 (0.05)	[0.84;0.85]	0.82 (0.09)	[0.90;0.91]	1.52 (0.17)	[1.73;1.73]	0.003	**
	15	0.86 (0.35)	[0.75;1.58]	0.77 (0.10)	[0.87;0.91]	2.05 (0.25)	[2.17;2.43]	0.003	**
	20	0.78 (0.18)	[0.93;1.02]	1.16 (0.45)	[1.65;1.65]	1.48 (0.24)	[1.66;1.79]	0.012	*
KW of magnitude (duration fixed)		0.041 *		0.024 *		0.001 **			
*IL6* (FC)	3	1.76 (0.24)	[1.92;2.08]	0.46 (0.05)	[0.51;0.52]	0.92 (0.15)	[1.06;1.15]	0.001	**
	6	1.67 (0.14)	[1.78;1.83]	0.41 (0.03)	[0.43;0.46]	1.11 (0.17)	[1.17;1.39]	0.001	**
	10	1.01 (0.41)	[1.33;1.53]	0.57 (0.02)	[0.57;0.59]	1.21 (0.14)	[1.32;1.37]	0.018	*
	15	2.01 (0.80)	[2.96;3.11]	0.38 (0.03)	[0.39;0.42]	1.03 (0.14)	[1.14;1.21]	0.001	**
	20	1.19 (0.14)	[1.30;1.38]	0.50 (0.10)	[0.56;0.67]	0.92 (0.14)	[1.00;1.06]	0.001	**
KW of magnitude (duration fixed)		<0.001 ***		<0.001 ***		0.036 *			
*RUNX2* (FC)	3	2.49 (0.20)	[2.65;2.78]	0.77 (0.07)	[0.83;0.83]	0.70 (0.12)	[0.84;0.87]	0.003	**
	6	2.15 (0.26)	[2.38;2.48]	0.80 (0.03)	[0.82;0.85]	0.89 (0.16)	[1.08;1.10]	0.003	**
	10	1.60 (0.84)	[2.37;2.44]	0.63 (0.04)	[0.67;0.69]	1.04 (0.18)	[1.25;1.27]	0.064	n.s.
	15	2.08 (0.15)	[2.22;2.30]	0.85 (0.04)	[0.86;0.92]	0.82 (0.07)	[0.86;0.92]	0.003	**
	20	1.32 (0.16)	[1.44;1.54]	0.81 (0.17)	[0.98;1.03]	0.62 (0.21)	[0.85;0.90]	0.002	**
KW of magnitude (duration fixed)		<0.001 ***		<0.001 ***		0.002 **			
*SP7* (FC)	3	2.18 (0.64)	[2.76;2.97]	2.75 (1.61)	[4.17;4.29]	0.50 (0.08)	[0.54;0.60]	0.003	**
	6	4.84 (2.33)	[6.56;6.94]	2.96 (1.24)	[3.94;4.17]	1.20 (1.32)	[2.07;3.53]	0.018	*
	10	4.22 (3.01)	[7.70;8.07]	1.56 (0.74)	[2.42;2.51]	1.84 (0.70)	[2.22;2.92]	0.140	n.s.
	15	4.34 (1.38)	[4.18;7.11]	3.20 (2.12)	[5.36;5.51]	2.37 (0.61)	[2.42;3.48]	0.059	n.s.
	20	0.65 (0.13)	[0.67;0.88]	0.84 (0.35)	[1.25;1.27]	0.81 (0.46)	[0.92;1.54]	0.519	n.s.
KW of magnitude (duration fixed)		<0.001 ***		0.022 *		0.002 **			
PGE2 (pg/well)	0	938.7 (80.1)	[1002.1;1002.6]	603.7 (16.6)	[615.3;629.6]	498.3 (32.3)	[526.3;542.6]	0.001	**
	3	1318.5 (166.3)	[1437.6;1477.3]	1165.5 (38.0)	[1197.8;1198.7]	1075.0 (67.1)	[1130.2;1131.5]	0.034	*
	6	1246.0 (83.8)	[1331.5;1348.9]	1080.2 (56.9)	[1110.2;1171.1]	934.5 (135.6)	[963.4;1195.3]	0.005	**
	10	1466.3 (143.4)	[1590.9;1591.6]	1096.7 (30.4)	[1130.2;1134.0]	972.2 (103.3)	[1093.4;1099.7]	0.002	**
	15	1625.5 (86.0)	[1642.1;1769.5]	1451.6 (190.9)	[1678.3;1709.5]	1240.2 (81.0)	[1321.7;1358.0]	0.006	**
	20	1962.5 (270.1)	[2140.6;2236.1]	1779.1 (96.5)	[1856.2;1885.7]	1456.2 (73.6)	[1505.3;1556.1]	0.003	**
KW of magnitude (duration fixed)		<0.001 ***		<0.001 ***		<0.001 ***			
IL6 (pg/well)	0	133.5 (20.6)	[157.1;159.8]	143.1 (13.4)	[147.2;166.8]	277.7 (35.1)	[305.1;312.5]	0.002	**
	3	308.6 (60.4)	[381.1;387.4]	300.5 (21.7)	[325.2;327.9]	512.4 (28.6)	[532.1;559.9]	0.003	**
	6	179.0 (12.1)	[184.6;198.8]	192.0 (24.5)	[211.7;216.8]	488.1 (71.1)	[549.0;563.2]	0.003	**
	10	203.4 (34.7)	[241.5;250.6]	186.3 (16.0)	[200.0;201.7]	335.0 (18.5)	[350.6;357.6]	0.003	**
	15	176.3 (4.5)	[179.5;180.0]	208.1 (8.4)	[215.1;220.0]	502.5 (46.2)	[541.5;574.6]	0.001	**
	20	316.1 (53.9)	[375.1;387.3]	291.3 (22.0)	[303.4;324.9]	506.0 (30.3)	[532.7;539.2]	0.003	**
KW of magnitude (duration fixed)		<0.001 ***		<0.001 ***		<0.001 ***			

* *P*_adj._ < 0.05; ** *P*_adj._ < 0.01; *** *P*_adj._ < 0.001; n.s., not significant.

**Table 2:** Cap parameters and media volume.

Parameter	Membrane Area Increase					
	0% (Control)	3%	6%	10%	15%	20%
Radius r (mm) ^1^	n.a.	50.62	36.82	29.54	25.25	22.82
Height h (mm) ^1^	n.a.	2.94	4.16	5.38	6.58	7.60
Volume of medium (mL)	2.89	4.37	4.96	5.54	6.08	6.52

^1^ Nazet et al. [55], Figure 1 with b = 17.0 mm.

**Table 3:** Specification of the PCR primers used for gene quantification.

Gene	GenBank Accession Number	Primer Sequence (f: 5′-Forward Primer-3′; r: 5′-Reverse Primer-3′)	Annealing Temp. (°C)	Data Acquisition Temp. (°C)	Amplicon Size (bp)	Primer Efficiency	Source
*PTGS2*	NM_000963.4	f: AAGCCTTCTCTAACCTCTCC r: GCCCTCGCTTATGATCTGTC	58	77	234	1.995	Janjic Rankovic et al. [14], Shi et al. [87]
*IL6*	NM_000600.5	f: TGGCAGAAAACAACCTGAACC r: TGGCTTGTTCCTCACTACTCTC	58	76	168	1.955	Janjic Rankovic et al. [14], Shi et al. [87]
*FOS*	NM_005252.4	f: GCTTTGCAGACCGAGATTGC r: TTGAGGAGAGGCAGGGTGAA	58	83	203	1.860	Janjic Rankovic et al. [14]
*RUNX2*	NM_001015051.4	f: GCGCATTCCTCATCCCAGTA r: GGCTCAGGTAGGAGGGGTAA	58	81	176	1.954	Shi et al. [7], Janjic Rankovic et al. [14]
*SP7*	NM_001173467.3	f: GGCACAAAGAAGCCGTACTC r: CACTGGGCAGACAGTCAGAA	61	81	247	1.935	Gronthos et al. [88]
*ALPL*	NM_001127501.4	f: GGACCATTCCCACGTCTTCAC r: CCTTGTAGCCAGGCCCATTG	64	80	137	1.968	Liu et al. [26]
*BGLAP*	NM_199173.6	f: AGCGAGGTAGTGAAGAGAC r: GAAAGCCGATGTGGTCAG	64	82	142	2.076	Gartland et al. [89]
*TNFRSF11B*	NM_001066	f: TCAAGCAGGAGTGCAATCG r: AGAATGCCTCCTCACACAGG	64	81	342	1.941	Yang et al. [46]
*TNF*	NM_000594.4	Commercial primer pair from realtimeprimers.com (Order information: VHPS-9415 ^†^)	58	79	173	1.967	Janjic Rankovic et al. [14], Shi et al. [87]
*POLR2A*	NM_000937.5	f: TCGCTTACTGTCTTCCTGTTGG r: TGTGTTGGCAGTCACCTTCC	58	79	108	1.886	Nazet et al. [55]
*RPL22*	NM_000983.4	f: TGATTGCACCCACCCTGTAG r: GGTTCCCAGCTTTTCCGTTC	61	75	98	1.939	Nazet et al. [55]

^†^ Real Time Primers, LLC, Elkins Park, PA, USA (primer sequences are disclosed upon purchase).

## Data Availability

All authors confirm that all related data supporting the findings of this study are given in the article and its Supplementary Materials.

## Supplementary Materials

The following are available online at www.mdpi.com/article/10.3390/ijms23031525/s1.

## Abbreviations

ALPL	Alkaline phosphatase, biomineralization associated
BGLAP	Bone gamma-carboxyglutamate protein
ELISA	Enzyme-linked immunosorbent assay
FC	Fold change
IL10	Interleukin 10
IL1B	Interleukin 1B
IL6	Interleukin 6
IL8	Interleukin 8
KW	Kruskal-Wallis test
MIQE	Minimum Information for Publication of Quantitative Real-Time PCR Experiment
OPG	Osteoprotegerin
OTM	Orthodontic teeth movement
*P*_adj._	Adjusted *P*
PDL	Periodontal ligament
PDLCs	Periodontal ligament cells
PGE2	Prostaglandin E2
PTGS2	Prostaglandin-endoperoxide synthase 2
qPCR	Quantitative real-time polymerase chain reaction
RANKL	Receptor activator of the nuclear factor kappa ligand
RT-qPCR	Reverse transcription qPCR
RUNX2	*Runt*-related transcription factor 2
SD	Standard deviation
TNF	Tumor necrosis factor α
TNFRSF11B	Tumor necrosis factor-alpha receptor superfamily member 11B
