# Supplementary material for: Effect of Different Parameters of In Vitro Static Tensile Strain on Human Periodontal Ligament Cells Simulating the Tension Side of Orthodontic Tooth Movement
Source: Int J Mol Sci. 2022 Jan 28;23(3):1525. doi: 10.3390/ijms23031525 (PMC8835937; doi:10.3390/ijms23031525)

Supplement 1

To manuscript

"Effect of different parameters of in vitro static tensile strain on human periodontal ligament cells simulating the tension side of orthodontic tooth movement"

Reference gene selection with RefFinder

URL: https://www.heartcure.com.au/reffinder/ (30-03-2021)

References

1. **BestKeeper**: Pfaffl MW, Tichopad A, Prgomet C, Neuvians TP. 2004. Determination of stable housekeeping genes, differentially regulated target genes and sample integrity: BestKeeper--Excel-based tool using pair-wise correlations. Biotechnology Letters 26:509-515.
2. **NormFinder**: Andersen CL, Jensen JL, Orntoft TF. 2004. Normalization of real-time quantitative reverse transcription-PCR data: a model-based variance estimation approach to identify genes suited for normalization, applied to bladder and colon cancer data sets. Cancer Research 64:5245-5250.
3. **Genorm**: Vandesompele J, De Preter K, Pattyn F, Poppe B, Van Roy N, De Paepe A, Speleman F. 2002. Accurate normalization of real-time quantitative RT-PCR data by geometric averaging of multiple internal control genes. Genome Biology 3:RESEARCH0034.
4. **The comparative delta-Ct method**: Silver N, Best S, Jiang J, Thein SL. 2006. Selection of housekeeping genes for gene expression studies in human reticulocytes using real-time PCR. BMC Molecular Biology 7:33.

Contents

Supplementary Table S1.1: Raw data (Cq) 2

Supplementary Table S1.2: Summary table of RefFinder results........ 3

Supplementary Table S1.3: Comprehensive gene stability........ 3

Supplementary Table S1.4: Gene stability by Delta CT method 4

Supplementary Table S1.5: Gene stability by BestKeeper 5

Supplementary Table S1.6: Gene stability by normFinder........ 6

Supplementary Table S1.7: Gene stability by Genorm 7

Supplementary Table S1.1: Raw data (Cq)

#	Sample type	Day	GAPDH	YWHAZ	PPIB	RPL0	RPL22	EEF1A1	POLR2A	RNA18S5
1	10%	1	18.94	21.14	21.55	22.02	21.85	18.39	22.71	7.89
2	10%	1	19.89	22.03	21.92	22.57	22.55	19.14	23.59	9.05
3	10%	1	19.03	21.26	21.72	21.93	21.74	18.51	22.53	8.16
4	20%	1	19.90	22.04	22.19	22.67	22.67	19.28	23.35	8.98
5	20%	1	19.51	21.72	21.96	22.23	22.10	18.86	22.96	8.50
6	20%	1	19.36	21.47	22.14	22.07	21.97	18.71	22.99	8.41
7	control	1	19.57	21.88	21.96	21.90	22.01	18.63	23.02	8.70
8	control	1	19.83	22.02	22.39	22.72	22.51	19.11	23.32	8.92
9	control	1	19.47	21.58	21.79	22.03	21.96	18.67	22.90	8.39
10	10%	3	18.55	21.51	20.65	20.81	21.01	16.96	22.66	7.69
11	10%	3	18.64	21.37	20.60	20.57	21.00	16.88	22.71	7.63
12	10%	3	18.80	21.60	20.92	21.55	21.66	17.57	22.88	7.76
13	20%	3	19.05	21.89	21.30	21.93	22.02	17.98	22.93	8.36
14	20%	3	19.26	22.13	22.04	21.46	21.81	17.88	23.23	8.87
15	20%	3	18.53	21.52	20.56	21.07	21.36	17.36	22.73	8.16
16	control	3	18.96	21.31	20.76	20.83	20.99	16.94	22.63	7.50
17	control	3	19.07	21.78	21.26	21.93	21.67	17.71	22.65	7.74
18	control	3	19.63	22.10	21.14	21.26	21.35	17.48	23.42	8.60

	Cq Values			
	**Control (N=6)**	**10 % (N=6)**	**20 % (N=6)**	**All (N=18)**
EEF1A1				
Mean (SD)	18.1 (0.838)	17.9 (0.915)	18.3 (0.720)	18.1 (0.799)
Median [Min; Max]	18.2 [16.9; 19.1]	18.0 [16.9; 19.1]	18.3 [17.4; 19.3]	18.2 [16.9; 19.3]
GAPDH				
Mean (SD)	19.4 (0.338)	19.0 (0.483)	19.3 (0.460)	19.2 (0.448)
Median [Min; Max]	19.5 [19.0; 19.8]	18.9 [18.6; 19.9]	19.3 [18.5; 19.9]	19.2 [18.5; 19.9]
POLR2A				
Mean (SD)	23.0 (0.331)	22.8 (0.381)	23.0 (0.223)	23.0 (0.310)
Median [Min; Max]	23.0 [22.6; 23.4]	22.7 [22.5; 23.6]	23.0 [22.7; 23.4]	22.9 [22.5; 23.6]
PPIB				
Mean (SD)	21.6 (0.601)	21.2 (0.574)	21.7 (0.644)	21.5 (0.605)
Median [Min; Max]	21.5 [20.8; 22.4]	21.2 [20.6; 21.9]	22.0 [20.6; 22.2]	21.6 [20.6; 22.4]
RNA18S5				
Mean (SD)	8.31 (0.565)	8.03 (0.534)	8.55 (0.315)	8.30 (0.504)
Median [Min; Max]	8.50 [7.50; 8.92]	7.83 [7.63; 9.05]	8.46 [8.16; 8.98]	8.38 [7.50; 9.05]
RPL0				
Mean (SD)	21.8 (0.657)	21.6 (0.763)	21.9 (0.568)	21.8 (0.642)
Median [Min; Max]	21.9 [20.8; 22.7]	21.7 [20.6; 22.6]	22.0 [21.1; 22.7]	21.9 [20.6; 22.7]
RPL22				
Mean (SD)	21.7 (0.535)	21.6 (0.581)	22.0 (0.425)	21.8 (0.510)
Median [Min; Max]	21.8 [21.0; 22.5]	21.7 [21.0; 22.6]	22.0 [21.4; 22.7]	21.8 [21.0; 22.7]
YWHAZ				
Mean (SD)	21.8 (0.294)	21.5 (0.314)	21.8 (0.271)	21.7 (0.312)
Median [Min; Max]	21.8 [21.3; 22.1]	21.4 [21.1; 22.0]	21.8 [21.5; 22.1]	21.7 [21.1; 22.1]

Supplementary Table S1.2: Summary table of RefFinder results

Method	Ranking Order (Better--Good--Average)							
	**1**	**2**	**3**	**4**	**5**	**6**	**7**	**8**
Delta CT	*RPL22*	*GAPDH*	*RNA18S5*	*PPIB*	*RPL0*	*POLR2A*	*YWHAZ*	*EEF1A1*
BestKeeper	*POLR2A*	*YWHAZ*	*GAPDH*	*RPL22*	*RNA18S5*	*RPL0*	*PPIB*	*EEF1A1*
Normfinder	*RPL22*	*GAPDH*	*RNA18S5*	*PPIB*	*RPL0*	*POLR2A*	*YWHAZ*	*EEF1A1*
Genorm	*YWHAZ | POLR2A*		*GAPDH*	*RNA18S5*	*RPL22*	*PPIB*	*RPL0*	*EEF1A1*
Recommended comprehensive ranking	RPL22	GAPDH	*POLR2A*	*YWHAZ*	*RNA18S5*	*PPIB*	*RPL0*	*EEF1A1*

Supplementary Table S1.3: Comprehensive gene stability

Genes	Geomean of ranking values
RPL22	2.11
GAPDH	2.45
POLR2A	2.45
YWHAZ	3.15
RNA18S5	3.66
PPIB	5.09
RPL0	5.69
EEF1A1	8.00

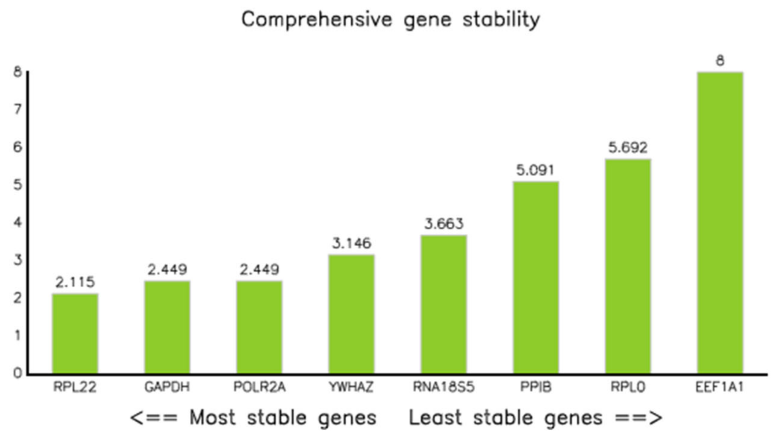

Supplementary Table S1.4: Gene stability by Delta CT method

**Genes**	**Average of STDEV**
RPL22	0.33
GAPDH	0.35
RNA18S5	0.36
PPIB	0.39
RPL0	0.40
POLR2A	0.41
YWHAZ	0.45
EEF1A1	0.49

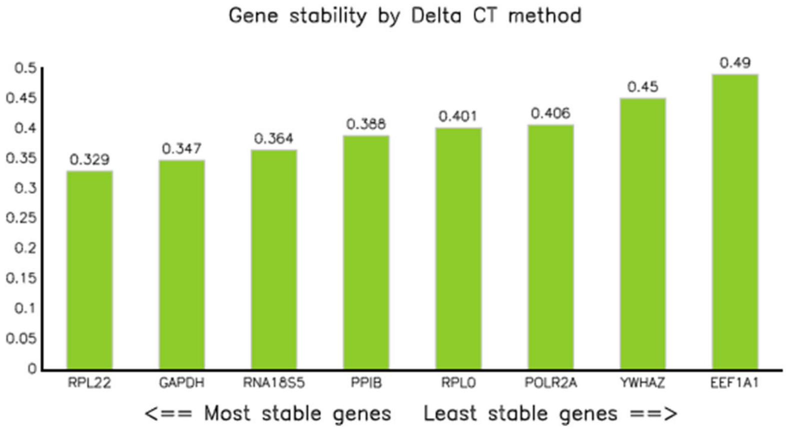

Supplementary Table S1.5: Gene stability by BestKeeper

CP data of housekeeping Genes by BEST KEEPER								
	GAPDH	*YWHAZ*	*PPIB*	*RPL0*	*RPL22*	*EEF1A1*	*POLR2A*	*RNA18S5*
n	18	18	18	18	18	18	18	18
geo Mean [CP]	19.22	21.68	21.48	21.74	21.78	18.10	22.95	8.28
AR Mean [CP]	19.22	21.69	21.49	21.75	21.79	18.11	22.96	8.30
min [CP]	18.53	21.14	20.56	20.57	20.99	16.88	22.53	7.50
max [CP]	19.90	22.13	22.39	22.72	22.67	19.28	23.59	9.05
std dev [+/- CP]	0.38	0.27	0.53	0.52	0.39	0.70	0.25	0.43
CV [% CP]	1.98	1.24	2.45	2.41	1.81	3.85	1.08	5.13
min [x-fold]	-1.61	-1.46	-1.90	-2.26	-1.73	-2.33	-1.34	-1.72
max [x-fold]	1.61	1.36	1.87	1.97	1.85	2.27	1.55	1.70
std dev [+/- x-fold]	1.30	1.20	1.44	1.44	1.31	1.62	1.19	1.34

Pearson correlation coefficient (r) by BEST KEEPER								
	GAPDH	*YWHAZ*	*PPIB*	*RPL0*	*RPL22*	*EEF1A1*	*POLR2A*	*RNA18S5*
YWHAZ	0.680	-	-	-	-	-	-	-
p-value	0.002	-	-	-	-	-	-	-
PPIB	0.827	0.450	-	-	-	-	-	-
p-value	0.001	0.061	-	-	-	-	-	-
RPL0	0.777	0.408	0.864	-	-	-	-	-
p-value	0.001	0.093	0.001	-	-	-	-	-
RPL22	0.802	0.529	0.870	0.966	-	-	-	-
p-value	0.001	0.024	0.001	0.001	-	-	-	-
EEF1A1	0.807	0.352	0.919	0.955	0.948	-	-	-
p-value	0.001	0.153	0.001	0.001	0.001	-	-	-
POLR2A	0.823	0.839	0.571	0.523	0.645	0.538	-	-
p-value	0.001	0.001	0.013	0.026	0.004	0.021	-	-
RNA18S5	0.851	0.772	0.797	0.695	0.805	0.766	0.865	-
p-value	0.001	0.001	0.001	0.001	0.001	0.001	0.001	-

Pearson correlation coefficient (r)								
**BestKeeper vs.**	*GAPDH*	*YWHAZ*	*PPIB*	*RPL0*	*RPL22*	*EEF1A1*	*POLR2A*	*RNA18S5*
coeff. of corr. [r]	0.919	0.660	0.920	0.898	0.947	0.928	0.784	0.931
p-value	0.001	0.003	0.001	0.001	0.001	0.001	0.001	0.001

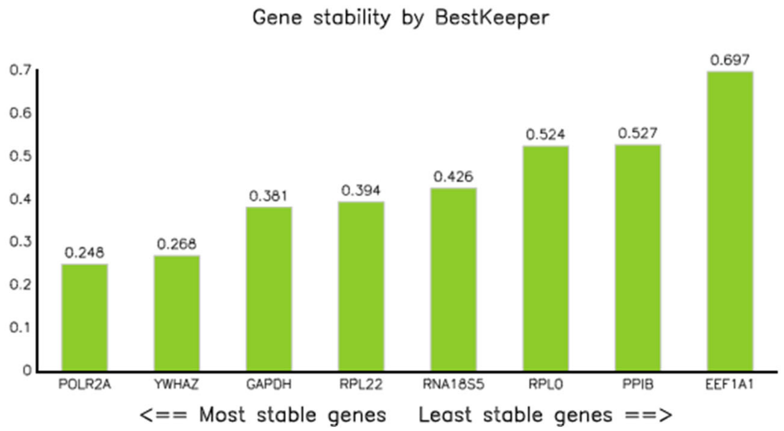

Supplementary Table S1.6: Gene stability by normFinder

Gene name	Stability value
RPL22	0.119
GAPDH	0.174
RNA18S5	0.216
PPIB	0.259
RPL0	0.301
POLR2A	0.327
YWHAZ	0.394
EEF1A1	0.442

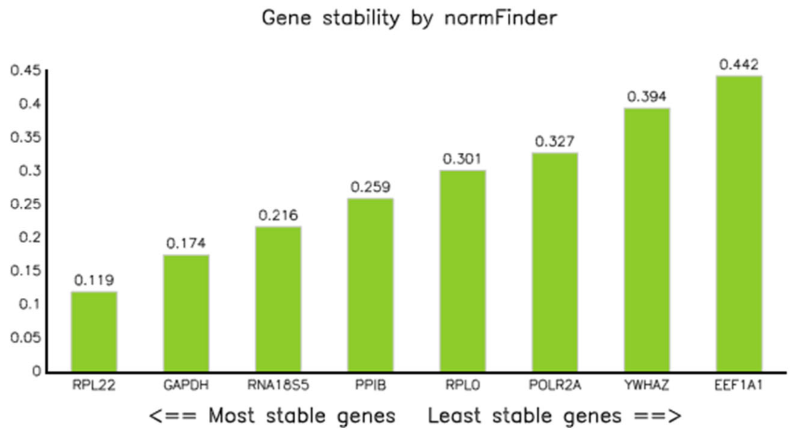

Supplementary Table S1.7: Gene stability by Genorm

Gene name	Stability value
YWHAZ | POLR2A	0.176
GAPDH	0.255
RNA18S5	0.274
RPL22	0.309
PPIB	0.343
RPL0	0.366
EEF1A1	0.397

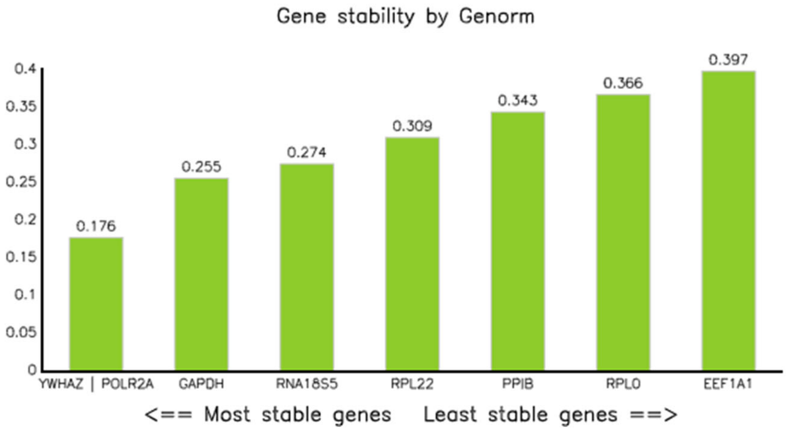

Supplement: Supplementary file 1 [file ijms-23-01525-s001.zip › Supplement_1_RefFinder_Results_updated.pdf]
