# Supplementary material for: Effect of Different Parameters of In Vitro Static Tensile Strain on Human Periodontal Ligament Cells Simulating the Tension Side of Orthodontic Tooth Movement
Source: Int J Mol Sci. 2022 Jan 28;23(3):1525. doi: 10.3390/ijms23031525 (PMC8835937; doi:10.3390/ijms23031525)
Supplement: Supplementary file 1 [file ijms-23-01525-s001.zip › Supplement_2_MIQE_checklist_and_primer_checking_updated.pdf]

Supplement 2

To the manuscript

"Effect of different parameters of in vitro static tensile strain on human periodontal ligament cells simulating the tension side of orthodontic tooth movement"

Contents

Supplementary Table S2.1: MIQE checklist for the RT-qPCR workflow 2

Supplementary Table S2.2: In-silico analysis of the RT-qPCR primer................................... 5

Supplementary Table S2.3: Primer validation by RT-qPCR.. 7

Supplementary Table S2.1: MIQE checklist for the RT-qPCR workflow

Reference: Bustin et al. (2010). BMC Mol Biol; 11:74.

	Details	Checklist
**Sample/Template**		
Source	If cancer, was biopsy screened for adjacent normal tissue?	Human periodontal ligament cells were isolated from health teeth using the explant technique described by Somerman et al. (1988) as previously published (Janjic Rankovic et al. 2020; Shi et al. 2019a; Shi et al. 2019b)
Method of preservation	Liquid N2/RNAlater/formalin	Preserved in liquid nitrogen
Storage time (if appropriate)	If using samples >6 months old	12 months
Handling	Fresh/frozen/formalin	Cell lysates were prepared using RNA lysis buffer from Quick-RNA™ MicroPrep kit (R1051; Zymo). After directly frozen in liquid nitrogen, cell lysates were stored at -80°C until further use for RNA extraction.
Extraction method	TriZol/columns	Defrosted cells lysates were passed through QIAshredder™ columns (Qiagen) to shear genomic DNA. The Quick-RNA™ Miniprep Kit (Zymo) was used for further RNA purification. After primary column purification, DNase I digestion was applied to reduce genomic DNA contamination as described by the manufacturer (Zymo). Finally, DNase/RNase-free water was used to elute the RNA from the columns. Before storage in the -80°C, RNase inhibitor RNasin® (Promega) was added to each preparation.
RNA:DNA-free	Intron-spanning primers/no RT control	Most primers were intron-spanning (Supplementary Table 2.2). Treatment with QIAshredder™ columns (Qiagen) and DNase I (Zymo) digestion were applied to reduce genomic DNA contamination. RT- (no RT) controls were tested and showed no contamination of genomic DNA.
Concentration	Nanodrop/ribogreen/microfluidics	Purity and concentration of extracted RNA were detected photometrically (Nanodrop ND-1000; PeqLab). Ratio of $A_{260/280}$>1.8 was found, indicating free of protein contamination during RNA preparations.
RNA: integrity	Microfluidics/3':5' assay	No.
Inhibition-free	Method of testing	Serial dilution of cDNA; as shown in “Primer efficiency” in Table 3 of the manuscript.
**Assay optimisation/validation**		
Accession number	RefSeq XX_1234567	Table 3 of the manuscript; Supplementary Table S2.2
Amplicon details	Exon location, amplicon size	Supplementary Table S2.2
Primer sequence	Even if previously published	Table 3 of the manuscript; Supplementary Tables S2.2 and S2.3. The sequence of the purchased primers from Realtimeprimers.com was disclosed upon purchase.

	Details	Checklist
*Probe sequence**	Identify LNA or other substitutions	No probes were used.
In silico	BLAST/Primer-BLAST/m-fold	Primer-BLAST, UCSC In-Silico PCR, Beacon Designer Free Edition, Primer-Check, uMELT, UNAfold and ENSEMBL were used for *in silico* test.
empirical	Primer concentration/annealing temperature	The optimal annealing temperatures were first identified by gradient PCR (Biometra TProfessional Gradient; Biometra, Goettingen, Germany) and then were finalized by qPCR on Roche LightCycler® 480 (LC480). Optimal annealing temperatures are recorded in Table 3 of the manuscript.
Priming conditions	Oligo-dT/random/combination/target-specific	The SuperScript® IV First Strand Synthesis System (Invitrogen) was used for cDNA synthesis with random hexamers provided. For each cDNA synthesis reaction, 600 ng total RNA was used. Target-specific primers for qPCR were used after assessment, as shown in Table 3 of the manuscript.
PCR efficiency	Dilution curve	Information on serial dilutions and primer efficiency was summarized in Supplementary Table S2.3. For each gene, two technical replicates were used for each dilution for qPCR. For analysis of qPCR including the standard curves, LC480 software version 1.5.0.39 was used.
Linear dynamic range	Spanning unknown targets	The analysing software for qPCR appointed the linear dynamic range automatically.
Limits of detection	LOD detection/accurate quantification	The analysing software for qPCR appointed the LOD automatically.
Intra-assay variation	Copy numbers not Cq	Each gene was detected on one individual plate.
**RT/PCR**		
Protocols	Detailed description, concentrations, volumes	For real-time PCR, the Luminaris Color HiGreen qPCR Master Mix Kit (K0392; Thermo Fisher Scientific, Vilnius, Lithuania) was used to detect gene expression of *PTGS2*, *IL6*, *TNF*, *RANKL*, *RUNX2*, *SP7*, *ALPL*, and *BGLAP* using the LightCycler® 480 with LC480 software version 1.5.0.39 (both from Roche Molecular Diagnostics, Basel, Switzerland). According to the manufacturer' protocol, 2 µl diluted cDNA (1:5 with double distilled, sterile water), 0.6 µl gene-specific forward primer, 0.6 µl gene-specific reverse primers, 6.8 µl PCR water and 10 µl qPCR Mastermix were added for reaction. PCR reactions proceeded as follows: 2 min of pretreatment of Uracil-DNA glycosylase (UDG) at 50 °C, 10 min of initial denaturation at 95 °C and 45 cycles of amplifications. Each amplification was consisted of four steps: 15 s of denaturation at 95 °C, 30 s of specific annealing temperature for each primer pair, 30 s of elongation at 72 °C and 5 s of data acquisition at the given temperature. For each plate, both no template controls (NTC) and no RT (reverse transcriptase) controls were added. NTCs were included for detection of primer dimers, while no RT controls were assessed for genomic DNA contamination.

	Details	Checklist
Reagents	Supplier, Lot number	Primers for genes were either acquired commercially or synthesized using sequences from related literatures. Primers from related literatures were verified by *in silico* tests using related bioinformatic tools given in Supplementary Table S2.2. All primers were synthesized by Metabion GmbH (Planegg/Steinkirchen, Germany; Oligonucleotide Purification Cartridge OPC® purification) or purchased from realtimeprimers.com. Information on the kits used (Quick-RNA™ MicroPrep kit; SuperScript® IV First Strand Synthesis kit, Invitrogen; Luminaris Color HiGreen qPCR Master Mix Kit) were all given in the manuscript.
Duplicate RT	ΔCq	No, but two technical replicates were repeated for each biological replicate at minimum.
NTC	Cq & melt curves	Yes
NAC	ΔCq beginning:end of qPCR	No, as no probes were used.
Positive control	Inter-run calibrators	No, each gene was tested on one plate with all samples included.
**Data analysis**		
Specialist software	e.g., QBAsePlus	IBM SPSS Statistics 26 (IBM Corp., Armonk, NY, USA)
Statistical justification	e.g., biological replicates	For each force magnitude for every force duration, three biological replicates were used. Each biological replicate was repeated with two technical replicates, giving a total of 6 amplifications of qPCR.
Transparent, validated normalisation	e.g., GeNorm summary	After testing with RT-qPCR using cDNA from some samples and assessment with Reffinder, *RPL22* and *POLR2A* were proved to be most stable in this experiment among the panel of reference genes. Therefore, *RPL22* and *POLR2A* were used as reference genes for following analysis.

Supplementary Table S2.2: *In-silico* analysis of the RT-qPCR primer

**Official gene symbol**	**Reference sequence** (NCBI GenBank)	**5'-forward primer-3'** (length / T_m / %GC / max. ΔG hairpin & self-dimer / Self-comp./Self-3'-comp.)	**5'-reverse primer-3'** (length / T_m / %GC / max. ΔG hairpin & self-dimer / Self-comp./Self-3'-comp.)	**Primer location** (max. ΔG cross-dimer)	**Amplicon** (length, %GC, T_m, SSAT)	**Amplicon location** (bp of Start/Stop)	**Intron-spanning** (length, bp)	**In silico qPCR specificity**	**Variants targeted** (Transcript/ Splice)	**Primer sequence source**
**Genes of interest**										
ALPL	NM_001127501.4	GGACCATTCCCACGTCTTCAC (21 / 58.2°C / 57% / -1.3 & -3.3 / 4/0)	CCTTGTAGCCAGGCCCATTG (20 / 57.9°C / 60% / -1.3 & -4.4 / 5/3)	Exon 1 (0)	137bp, 55%, 90.5°C, No	1330 / 1466	Yes (597)	Yes (BLAST, UCSC)	Yes	(Liu et al. 2017)
BGLAP	NM_199173.6	AGCGAGGTAGTGAAGAGAC (19 / 52.6°C / 53% / n.f. & n.f. / 2/1)	GAAAGCCGATGTGGTCAG (18 / 52.3°C / 56% / n.f. & n.f. / 2/1)	Exon 3/4 (-1.1)	142bp, 61%, 93.5°C, No	175 / 316	Yes (201)	Yes (BLAST, UCSC)	Yes	(Gartland et al. 2005)
PTGS2	NM_000963.4	AAGCCTTCTCTAACCTCTCC (20 / 52.9°C / 50% / n.f. & -0.5 / 5/0)	GCCCTCGCTTATGATCTGTC (20 / 55.2°C / 55% / n.f. & -2.0 / 4/1)	Exon 4/5 (-2.9)	234bp, 45%, 88°C, No	510 / 743	Yes (430)	Yes (BLAST, UCSC)	Yes	(Janjic Rankovic et al. 2020; Shi et al. 2019a)
FOS	NM_005252.4	GCTTTGCAGACCGAGATTGC (20 / 57.2°C / 55% / -2.0 & -3.4 / 4/2)	TTGAGGAGAGGCAGGGTGAA (20 / 57.3°C / 55% / n.f. & n.f. / 2/0)	Exon 4 (-2.0)	203bp, 57%, 93.5°C, No	687 / 889	No	Yes (BLAST, UCSC)	Yes	(Janjic Rankovic et al. 2020)
IL6	NM_000600.5	TGGCAGAAAACAACCTGAACC (21 / 56.5°C / 48% / -1.1 & -1.1 / 3/0)	TGGCTTGTTCCTCACTACTCTC (22 / 56.9% / 50% / n.f. & n.f. / 2/0)	Exon 2/3 (-3.3)	168bp, 43%, 85.5°C, No	317 / 484	Yes (707)	Yes (BLAST, UCSC)	Yes	(Janjic Rankovic et al. 2020; Shi et al. 2019a)
RUNX2	NM_001015051.4	GCGCATTCCTCATCCCAGTA (20 / 56.9°C / 55% / n.f. & -5.2 / 4/2)	GGCTCAGGTAGGAGGGGTAA (20 / 56.9°C / 60% / -1.0 & -1.0 / 3/1)	Exon 6/7 (-2.9)	176bp, 57%, 92.0°C, No	947 / 1122	Yes (20131)	Yes (BLAST, UCSC)	Yes	(Janjic Rankovic et al. 2020; Shi et al. 2019b)
SP7	NM_001173467.3	GGCACAAAGAAGCCGTACTC (20 / 56.2°C / 55% / -2.4 & -2.4 / 4/0)	CACTGGGCAGACAGTCAGAA (20 / 56.6°C / 55% / -2.5 & -2.5 / 5/1)	Exon 3 (-2.4)	247bp, 57%, 93°C, No	383 / 629	No	Yes (BLAST, UCSC)	Yes	(Gronthos et al. 2003)
TNFRSF11B	NM_002546.4	TCAAGCAGGAGTGCAATCG (19 / 54.9°C / 53% / -2.0 & -3.4 / 6/4)	AGAATGCCTCCTCACACAGG (20 / 56.3°C / 55% / -1.3 & -1.3 / 4/1)	Exon 2/4 (-4.1)	342bp, 46%, 88.5°C/91°C, Yes	342 / 683	Yes (6020)	Yes (BLAST, UCSC)	Yes	(Yang et al. 2010)
TNF	NM_000594.4	Commercial primer (20 / 55.9°C / 55% / n.f. & n.f. / 3/0)	Commercial primer (20 / 54.2°C / 45% / n.f. & -4.4 / 6/2)	Exon 4 (-2.0)	173bp, 50%, 85.5°C, Yes	Commercial primer from realtimeprimers.com (Order information: VHPS-9415)	No	Yes (BLAST, UCSC)	Yes	Realtimeprimers.com; (Janjic Rankovic et al. 2020; Shi et al. 2019a)
**Reference genes**										
EEF1A1	NM_001402.6	CCTGCCTCTCCAGGATGTCTAC (22 / 59.0°C / 59% / -3.0 & -3.0 / 5/2)	GGAGCAAAGGTGACCACCATAC (22 / 58.7°C / 55% / -1.5. & -3.2 / 6/2)	Exon 5/6 (–2.9)	105bp, 52%, 88°C, No	804 / 908	Yes (87)	Yes (BLAST/UCSC)	Yes	(Nazet et al. 2020)
GAPDH	NM_002046.7	CTCCTGTTCGACAGTCAGCC (20 / 57.4°C / 60% / -2.5 & -3.1 / 6/1)	CGACCAAATCCGTTGACTCC (20 / 55.9°C/ 55%/ -0.7 & -0.7 / 3/1)	Exon 1 / 2-3 (–3.8)	103bp, 58%, 91°C, No	12 / 114	Yes, rev. primer on exon junction	Yes (BLAST/UCSC)	Yes	(Chirieleison et al. 2017)
POLR2A	NM_000937.5	TCGCTTACTGTCTTCCTGTTGG (22 / 57.8°C / 50% / n.f. & n.f. / 3/0)	TGTGTTGGCAGTCACCTTCC (20 / 57.4°C / 55% / -1.3 & -1.3 / 3/3)	Exon 21/22 (–2.5)	108bp, 53%, 89.5°C, No	3811 / 3918	Yes (468)	Yes (BLAST/UCSC)	Yes	(Nazet et al. 2020)
PPIB	NM_000942.5	TTCCATCGTGTAATCAAGGACTTC (24 / 56.7°C / 42% / -1.3 & -1.3 / 4/2)	GCTCACCGTAGATGCTCTTTC (21 / 56.1°C / 52% / -0.7 & -0.7 / 4/0)	Exon 3/4 (–2.1)	88bp, 53%, 87°C, No	313 / 400	Yes (3194)	Yes (BLAST/UCSC)	Yes	(Nazet et al. 2020)
RNA18SN5	NR_003286.4	AACTGCGAATGGCTCATTAAATC (23 / 55.8°C / 39% / -1.7 & -1.7 / 6/3)	GCCCGTCGGCATGTATTAG (19 / 55.2°C / 58% / -2.4 & -2.4 / 5/1)	n.a. (–2.4)	103bp, 46%, 85.5°C, No	84 / 186	No (rRNA)	No (RNA45S5 also targeted)	—	(Nazet et al. 2020)

Official gene symbol	Reference sequence (NCBI GenBank)	5'-forward primer-3' (length / T_m / %GC / max. ΔG hairpin & self-dimer / Self-comp./Self-3'-comp.)	5'-reverse primer-3' (length / T_m / %GC / max. ΔG hairpin & self-dimer / Self-comp./Self-3'-comp.)	Primer location (max. ΔG cross-dimer)	Amplicon (length, %GC, T_m, SSAT)	Amplicon location (bp of Start/Stop)	Intron-spanning (length, bp)	In silico qPCR specificity	Variants targeted (Transcript/ Splice)	Primer sequence source
RPL0	NM_001002.4	GAAACTCTGCATTCTCGCTTCC (22 / 57.4°C / 50% / -0.6 & -3.4 / 4/0)	GACTCGTTTGTACCCGTTGATG (22 / 57.1°C / 50% / n.f. & -2.0 / 4/0)	Exon 6/7 (–1.8)	120bp, 50%, 89°C, No	702 / 821	Yes (1091)	Yes (BLAST/UCSC)	Yes	(Nazet et al. 2020)
RPL22	NM_000983.4	TGATTGCACCCACCCTGTAG (20 / 56.6°C / 55% / n.f. & -3.4 / 4/2)	GGTTCCCAGCTTTTCCGTTC (20 / 56.4°C / 55% / n.f. & -3.0 / 4/0)	Exon 2/3 (–1.5)	98bp, 44%, 84°C, No	91 / 188	Yes (4597)	Yes (BLAST/UCSC)	Yes	(Nazet et al. 2020)
YWHAZ	NM_003406.4	AGGAGATTACTACCGTTACTTGGC (24 / 57.8°C / 46% / n.f. & n.f. / 4/2)	AGCTTCTTGGTATGCTTGTTGTG (23 / 57.4°C / 43% / -1.8 & -3.0 / 4/0)	Exon 8/9 (–2.2)	91bp, 47%, 86°C, No	491 / 581	Yes (617)	Yes (BLAST/UCSC)	Yes	(Nazet et al. 2020)

T_m, melting temperature of primer or qPCR product (amplicon); %GC, percent of guanin/cytosine content; bp, base pairs; max. ΔG hairpin, maximal ΔG of hairpin; max. ΔG self-dimer, maximal ΔG of self-dimer; Self-comp., self complementary; Self-3'-comp., self 3' complementary; max. ΔG Cross-dimer, maximal ΔG of cross-dimer; SSAT, secondary structures at annealing temperature (at primer binding sites); n.f., not found.

To perform silico analysis of RT-qPCR primers, their targets and corresponding amplification products, the following programs and online resources were used. All URLs were valid on 02-12-2020.

a. Primer-BLAST (URL: https://www.ncbi.nlm.nih.gov/tools/primer-blast/) was used to check the "length" of primer, "In silico qPCR specificity", possible co-amplification of genomic DNA, "Self-comp." and "Self-3'-comp".
b. UCSC In-Silico PCR (URL: https://genome.ucsc.edu/cgi-bin/hgPcr) was used to check "In silico qPCR specificity" and RT-qPCR in genomic context.
c. "Amplicon (length)", "Amplicon location (bp of Start/Stop)", "Intron-spanning (length)" was identified or calculated by either Primer-BLAST or UCSC In-Silico PCR
d. Beacon Designer™ Free Edition (Premier BioSoft International, Palo Alto, CA, USA, URL: http://www.premierbiosoft.com/qOligo/Oligo.jsp?PID=1) was used to identify primers' specifications (T_m, %GC, max. ΔG hairpin & max. ΔG self-dimer, max. ΔG cross-dimer).
e. Primer-Check (URL: http://projects.insilico.us/SpliceCenter/PrimerCheck.jsp) was used to check for "Primer location", which means the exon/intron binding sites.
f. uMelt (URL: https://dna-utah.org/umelt/quartz/) was used to check "Amplicon (%GC, Tm)", which means GC content and melting temperature of the amplicon.
g. UNAFold@IDT-DNA (URL: http://eu.idtdna.com/UNAFold?, maximum sequence length 255 bases) was used to check "Amplicon (SSAT)", to identify occurrence of secondary structures in the RT-qPCR product during the annealing step. mFold @ http://www.unafold.org/mfold/applications/dna-folding-form.php :
h. ENSEMBL (URL: https://www.ensembl.org) was used to check "Variants targeted (Transcript/Splice)".

Supplementary Table S2.3: Primer validation by RT-qPCR

Gene symbol	Primer sequence (f: 5'-forward primer-3'; r: 5'-reverse primer-3')	Primer sequence source	Prediluted cDNA (1:5) used?	Specificity by melting curve / T_m (°C)	Specificity by agarose gel / amplicon size (bp)	Annealing temp. (°C)/ data acquisition temp. (°C)	Dilution series used for efficiency testing	Primer efficiency			
								Efficiency	Error	Slope	Y intercept
**Genes of interest**											
ALPL	f: GGACCATTCCCACGTCTTCAC<br>r: CCTTGTAGCCAGGCCCATTG	(Liu et al. 2017)	Undiluted	Yes / 82.3	Yes / 137	64 / 80	Undil., 1:4, 1:16, 1:64, 1:256, 1:1024	1.968	0.0291	-3.401	37.70
BGLAP	f: AGCGAGGTAGTGAAGAGAC<br>r: GAAAGCCGATGTGGTCAG	(Gartland et al. 2005)	Prediluted	Yes / 84.6	Yes / 142	64 / 82	Predil., 1:4, 1:16, 1:64, 1:256, 1:1024	2.076	0.00897	-3.152	35.71
PTGS2	f: AAGCCTTCTCTAACCTCTCC<br>r: GCCCTCGCTTATGATCTGTC	(Janjic Rankovic et al. 2020; Shi et al. 2019a)	Prediluted	Yes / 79.4	Yes / 234	58 / 77	Predil., 1:4, 1:16, 1:64, 1:256	1.995	0.0356	-3.335	35.95
FOS	f: GCTTTGCAGACCGAGATTGC<br>r: TTGAGGAGAGGCAGGGTGAA	(Janjic Rankovic et al. 2020)	Prediluted	Yes / 84.7	Yes / 203	58 / 83	Predil., 1:4, 1:16, 1:64, 1:256	1.860	0.0327	3.711	37.74
IL6	f: TGGCAGAAAACAACCTGAACC<br>r: TGGCTTGTTCCTCACTACTCTC	(Janjic Rankovic et al. 2020; Shi et al. 2019a)	Prediluted	Yes / 78.1	Yes / 168	58 / 76	Predil., 1:4, 1:16, 1:64, 1:256	1.955	0.0231	-3.434	39.18
RUNX2	f: GCGCATTCCTCATCCCAGTA<br>r: GGCTCAGGTAGGAGGGGTAA	(Janjic Rankovic et al. 2020; Shi et al. 2019b)	Prediluted	Yes / 83.1	Yes / 176	58 / 81	Predil., 1:4, 1:16, 1:64, 1:256, 1:1024	1.954	0.0129	-3.437	34.96
SP7	f: GGCACAAAGAAGCCGTACTC<br>r: CACTGGGCAGACAGTCAGAA	(Gronthos et al. 2003)	Undiluted	Yes / 84	Yes / 247	61 / 81	Undil., 1:4, 1:16, 1:32, 1:64, 1:128	1.935	0.0367	-3.489	36.28
TNFRSF11B	f: TCAAGCAGGAGTGCAATCG<br>r: AGAATGCCTCCTCACACAGG	(Yang et al. 2010)	Prediluted	Yes / 83	Yes / 342	64 / 81	Predil., 1:5, 1:25, 1:125, 1:625	1.941	0.0211	-3.473	32.63
TNF	Commercial primer pair from realtimeprimers.com (Order information: VHPS-9415)	(Janjic Rankovic et al. 2020; Shi et al. 2019a)	Undiluted	Yes / 81	Yes / 173	58 / 79	Undil., 1:4, 1:16, 1:32, 1:64, 1:128	1.967	0.0421	-3.404	37.27
**Reference genes**											
EEF1A1	f: CCTGCCTCTCCAGGATGTCTAC<br>r: GGAGCAAAGGTGACCACCATAC	(Nazet et al. 2020)	Prediluted	Yes / 79.9	Yes / 105	61 / 77	Predil., 1:10, 1:100, 1:1000, 1;10,000	1.981	0.00773	-3.367	30.06
GAPDH	f: CTCCTGTTCGACAGTCAGCC<br>r: CGACCAAATCCGTTGACTCC	(Chirieleison et al. 2017)	Prediluted	Yes / 82.4	Yes / 103	52 / 79	Predil., 1:10, 1:100, 1:1000, 1;10,000	1.970	0.00209	-3.396	31.55
POLR2A	f: TCGCTTACTGTCTTCCTGTTGG<br>r: TGTGTTGGCAGTCACCTTCC	(Nazet et al. 2020)	Prediluted	Yes / 81.9	Yes / 108	58 / 79	Predil., 1:10, 1:100, 1:1000, 1;10,000	1.886	0.0150	-3.630	37.25

Gene symbol	Primer sequence (f: 5'-forward primer-3'; r: 5'-reverse primer-3')	Primer sequence source	Prediluted cDNA (1:5) used?	Specificity by melting curve / T_m (°C)	Specificity by agarose gel / amplicon size (bp)	Annealing temp. (°C)/ data acquisition temp. (°C)	Dilution series used for efficiency testing	Primer efficiency			
								Effici-ency	Error	Slope	Y inter-cept
PPIB	f: TTCCATCGTGTAATCAAGGACTTC<br>r: GCTCACCGTAGATGCTCTTTC	(Nazet et al. 2020)	Prediluted	Yes / 80.1	Yes / 88	55 / 77	Predil., 1:10, 1:100, 1:1000, 1;10,000	1.909	0.00896	-3.560	35.93
RNA18S5	f: AACTGCGAATGGCTCATTAAATC<br>r: GCCCGTCGGCATGTATTAG	(Nazet et al. 2020)	Prediluted	Yes / 77.8	Yes / 103	55 / 55	Predil., 1:10, 1:100, 1:1000, 1;10,000	1.869	0.00242	-3.682	21.57
RPL0	f: GAAACTCTGCATTCTCGCTTCC<br>r: GACTCGTTTGTACCCGTTGATG	(Nazet et al. 2020)	Prediluted	Yes / 81.3	Yes / 120	64 / 79	Predil., 1:10, 1:100, 1:1000, 1;10,000	2.010	0.0430	-3.299	33.73
RPL22	f: TGATTGCACCCACCCTGTAG<br>r: GGTTCCCAGCTTTTCCGTTC	(Nazet et al. 2020)	Prediluted	Yes / 77.6	Yes / 98	61 / 75	Predil., 1:10, 1:100, 1:1000, 1;10,000	1.939	0.0160	-3.478	34.41
YWHAZ	f: AGGAGATTACTACCGTTACTTGGC<br>r: AGCTTCTTGGTATGCTTGTTGTG	(Nazet et al. 2020)	Prediluted	Yes / 78.9	Yes / 91	55 / 76	Predil., 1:10, 1:100, 1:1000, 1;10,000	1.945	0.0216	-3.462	34.89

References

Chirieleison SM, Marsh RA, Kumar P, Rathkey JK, Dubyak GR, Abbott DW (2017). Nucleotide-binding oligomerization domain (NOD) signaling defects and cell death susceptibility cannot be uncoupled in X-linked inhibitor of apoptosis (XIAP)-driven inflammatory disease. J Biol Chem; 292(23):9666-9679.

Gartland A, Buckley KA, Dillon JP, Curran JM, Hunt JA, Gallagher JA (2005). Isolation and Culture of Human Osteobalsts. In: Picot J (Ed.). Human Cell Culture Protocols. [Methods in Molecular Medicine; 107] 2nd Ed. Totowa, NJ: Humana Press; pp. 29-54.

Gronthos S, Zannettino AC, Hay SJ, Shi S, Graves SE, Kortesidis A, Simmons PJ (2003). Molecular and cellular characterisation of highly purified stromal stem cells derived from human bone marrow. J Cell Sci; 116(Pt 9):1827-35.

Janjic Rankovic M, Docheva D, Wichelhaus A, Baumert U (2020). Effect of static compressive force on in vitro cultured PDL fibroblasts: monitoring of viability and gene expression over 6 days. Clin Oral Investig; 24(7):2497-2511.

Liu J, Li Q, Liu S, Gao J, Qin W, Song Y, Jin Z (2017). Periodontal Ligament Stem Cells in the Periodontitis Microenvironment Are Sensitive to Static Mechanical Strain. Stem Cells Int; 2017:1380851.

Nazet U, Schröder A, Spanier G, Wolf M, Proff P, Kirschneck C (2020). Simplified method for applying static isotropic tensile strain in cell culture experiments with identification of valid RT-qPCR reference genes for PDL fibroblasts. Eur J Orthod; 42(4):359-370.

Shi J, Baumert U, Folwaczny M, Wichelhaus A (2019a). Influence of static forces on the expression of selected parameters of inflammation in periodontal ligament cells and alveolar bone cells in a co-culture in vitro model. Clin Oral Investig; 23(6):2617-2628.

Shi J, Folwaczny M, Wichelhaus A, Baumert U (2019b). Differences in RUNX2 and P2RX7 gene expression between mono- and coculture of human periodontal ligament cells and human osteoblasts under compressive force application. Orthod Craniofac Res; 22(3):168-176.

Somerman MJ, Archer SY, Imm GR, Foster RA (1988). A comparative study of human periodontal ligament cells and gingival fibroblasts *in vitro*. J Dent Res; 67(1):66-70.

Yang Y, Yang Y, Li X, Cui L, Fu M, Rabie AB, Zhang D (2010). Functional analysis of core binding factor a1 and its relationship with related genes expressed by human periodontal ligament cells exposed to mechanical stress. Eur J Orthod; 32(6):698-705.
